# ATRA mechanically reprograms pancreatic stellate cells to suppress matrix remodelling and inhibit cancer cell invasion

**DOI:** 10.1038/ncomms12630

**Published:** 2016-09-07

**Authors:** Antonios Chronopoulos, Benjamin Robinson, Muge Sarper, Ernesto Cortes, Vera Auernheimer, Dariusz Lachowski, Simon Attwood, Rebeca García, Saba Ghassemi, Ben Fabry, Armando del Río Hernández

**Affiliations:** 1 Cellular and Molecular Biomechanics Laboratory, Department of Bioengineering, Imperial College London, London SW7 2AZ, UK; 2 Department of Physics, Biophysics Group, University of Erlangen-Nuremberg, Erlangen 91054, Germany; 3 Department of Pathology and Laboratory Medicine, University of Pennsylvania School of Medicine, Philadelphia, Pennsylvania 19104, USA

## Abstract

Pancreatic ductal adenocarcinoma (PDAC) is a highly aggressive malignancy with a dismal survival rate. Persistent activation of pancreatic stellate cells (PSCs) can perturb the biomechanical homoeostasis of the tumour microenvironment to favour cancer cell invasion. Here we report that ATRA, an active metabolite of vitamin A, restores mechanical quiescence in PSCs via a mechanism involving a retinoic acid receptor beta (RAR-β)-dependent downregulation of actomyosin (MLC-2) contractility. We show that ATRA reduces the ability of PSCs to generate high traction forces and adapt to extracellular mechanical cues (mechanosensing), as well as suppresses force-mediated extracellular matrix remodelling to inhibit local cancer cell invasion in 3D organotypic models. Our findings implicate a RAR-β/MLC-2 pathway in peritumoural stromal remodelling and mechanosensory-driven activation of PSCs, and further suggest that mechanical reprogramming of PSCs with retinoic acid derivatives might be a viable alternative to stromal ablation strategies for the treatment of PDAC.

## Abstract (web summary)

Persistent activation of pancreatic stellate cells (PSCs) can perturb the biomechanical homeostasis of the tumour microenvironment. Here the authors show that all-trans retinoic acid reduces retinoic acid receptor beta dependent-actomyosin contractility and restores mechanical quiescence in PSCs.

## Graphical abstract

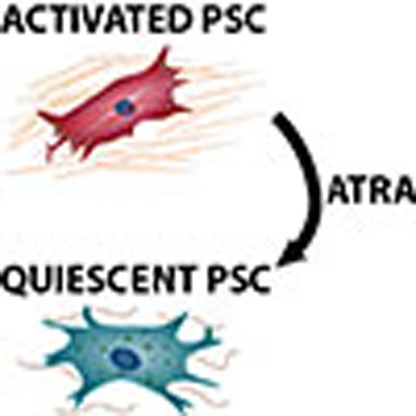

Pancreatic ductal adenocarcinoma (PDAC) is an extremely aggressive cancer with a dismal 5-year survival rate of 4% and a median survival of 6 months despite advances in conventional therapies targeting cancer cells^1^. PDAC is characterized by a strong desmoplastic reaction or stromal fibrosis, which is driven by pancreatic stellate cells (PSCs) and is believed to create a unique microenvironment that regulates tumour growth, metastasis and chemoresistance^2^,^3^,^4^. Recently, this desmoplastic reaction has been the focus of several studies that have emphasized the complex nature of the stromal components and their contribution to disease progression^5^,^6^,^7^,^8^,^9^,^10^,^11^.

In pancreatic cancer, PSCs transition from a quiescent, lipid-vitamin-A storing phenotype to an activated, myofibroblast-like phenotype that is accompanied by changes in their cytoskeletal and contractile activity, migratory capacity, extracellular matrix (ECM) synthesis and acquisition of an expansive secretome^12^. The contractile myofibroblast-like phenotype is a general hallmark feature of cancer-associated fibroblasts (CAFs)^13^. As in other conditions featuring pathological tissue fibrosis, myofibroblasts need to establish a mechanical feedback loop to perpetuate their fully activated state by promoting and sensing a stiff microenvironment. Annulment of this positive-feedback loop is sufficient to abrogate their activation^14^,^15^,^16^,^17^. This loop entails the cell capacity to (i) remodel and stiffen its microenvironment by applying endogenous cell-generated forces to the ECM and (ii) sense and respond to external mechanical stimuli from the ECM (also known as mechanosensing or reinforcement). Both properties critically depend on the cell's contractile actomyosin machinery^18^,^19^.

CAFs alter not only the biochemical milieu but also the biomechanical homoeostasis of the tumour microenvironment. CAFs use contractile forces or proteolytic activity to remodel the ECM to create tracks for migration of cancer cells^20^,^21^. Force-mediated matrix remodelling is dependent on actomyosin contractility generated through phosphorylation of the regulatory myosin light-chain 2 (MLC-2) and activation of myosin II. A high level of actomyosin contractility is crucial for the emergence, maintenance and functional activity of tumour-associated myofibroblasts^13^,^22^.

Stromal reprogramming, as opposed to ablation, is an emerging concept gaining acceptance in the realm of stroma-targeting approaches for the treatment of PDAC^23^. We hypothesized that retinoids could be well poised to reprogram the tumour stroma due to their pleiotropic mode of action and ability to regulate a large number of genes involved in CAF function. We report that all-trans retinoic acid (ATRA), an active metabolite of vitamin A, restores mechanical quiescence in PSCs through a previously unidentified mechanism involving a retinoic acid receptor beta (RAR-β)-dependent downregulation of actomyosin (MLC-2) contractility. We show that ATRA treatment reduces the ability of PSCs to generate high traction forces, adapt to extracellular mechanical cues and suppresses force-mediated ECM remodelling to inhibit local cancer cell invasion in three-dimensional (3D) organotypic models.

## Results

### ATRA increases focal adhesion size and cell–ECM adhesion

**Figure 1:**
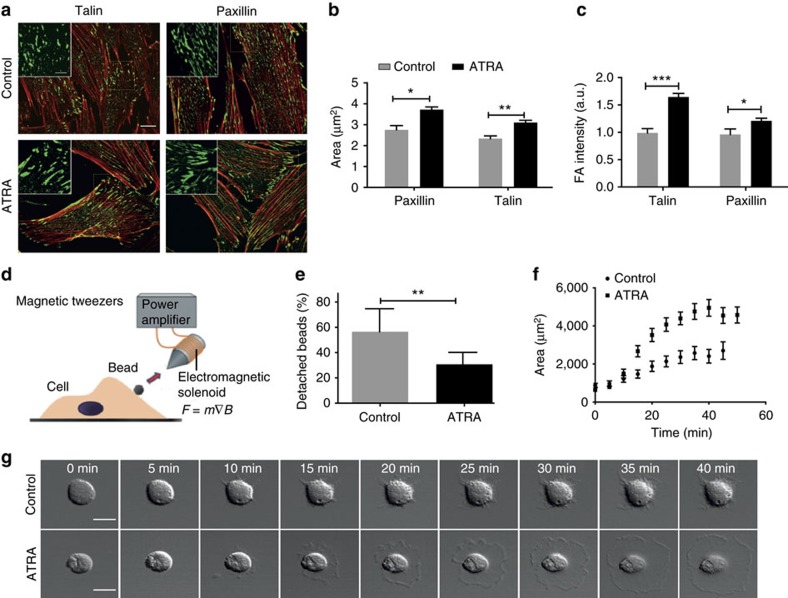
ATRA increases focal adhesion size and cell–ECM adhesion strength of PSCs. (**a**) Immunostaining images of talin and paxillin. Scale bar, 20 μm. Zoomed areas, 10 μm. (**b**,**c**) Quantification of normalized focal adhesion area and intensity. (**d**) Schematic representation of the magnetic tweezers device used in this study to apply forces on the cell surface. (**e**) Quantification of cell-bound beads that detached after force application. (**f**) Quantification of cell spreading area over time. (**g**) Time-elapsed sequential images of PSCs spreading until PSCs attained maximum area. Scale bars, 10 μm. In all panels, error bars represent s.e.m. *P<0.05; **P<0.01; ***P<0.001; (*t*-test); three experimental replicates.

The bidirectional mechanical communication between cells and the ECM is mediated by integrin-based focal adhesion complexes. These complexes connect the actin cytoskeleton with the extracellular protein ligands in the ECM, allowing cells to adhere to the ECM, transmit endogenous contractile forces and sense the ECM rigidity^24^. To investigate how ATRA treatment affects the ability of activated PSCs to promote and sense a stiff environment, and therefore to maintain their myofibroblasts phenotype, we first sought to characterize focal adhesion complexes. ATRA-treated PSCs displayed significantly larger and brighter focal adhesion complexes (both for talin and paxillin) relative to untreated control PSCs (Fig. 1a–c). To compare this result with the sizes of focal adhesions present in quiescent PSCs, we grew PSCs on matrigel for 10 days, a technique to induce quiescence^25^, and we used Oil Red staining to identify the lipid droplets characteristic of PSC quiescence (Supplementary Fig. 1). We observed that quiescent PSCs display larger focal adhesion in comparison with control PSC, and that ATRA-treated PSCs displayed significantly larger focal adhesions with respect to control activated PSCs and quiescent PSCs grown on matrigel. The assembly of larger focal adhesion complexes in ATRA-treated PSCs was positively associated with a higher matrix adhesion strength compared with control cells, whereby application of a pulling force of 1 nN generated by a magnetic tweezers device resulted in a significantly lower number (reduced by half) of fibronectin (FN)-coated magnetic beads detaching from the cell surface (Fig. 1d,e). We further characterized the rate of isotropic cell spreading with time-lapse video microscopy by measuring the spread area as a function of time until the cell attained its maximum spread area. ATRA-treated PSCs spread significantly faster relative to untreated PSCs and attained a higher maximum spread area (Fig. 1f,g). Taken together, these results indicate that ATRA-treated cells form larger focal adhesion complexes, spread faster, attain a larger spreading area and attach stronger to the ECM.

### ATRA reduces endogenous force generation and cell stiffness

**Figure 2:**
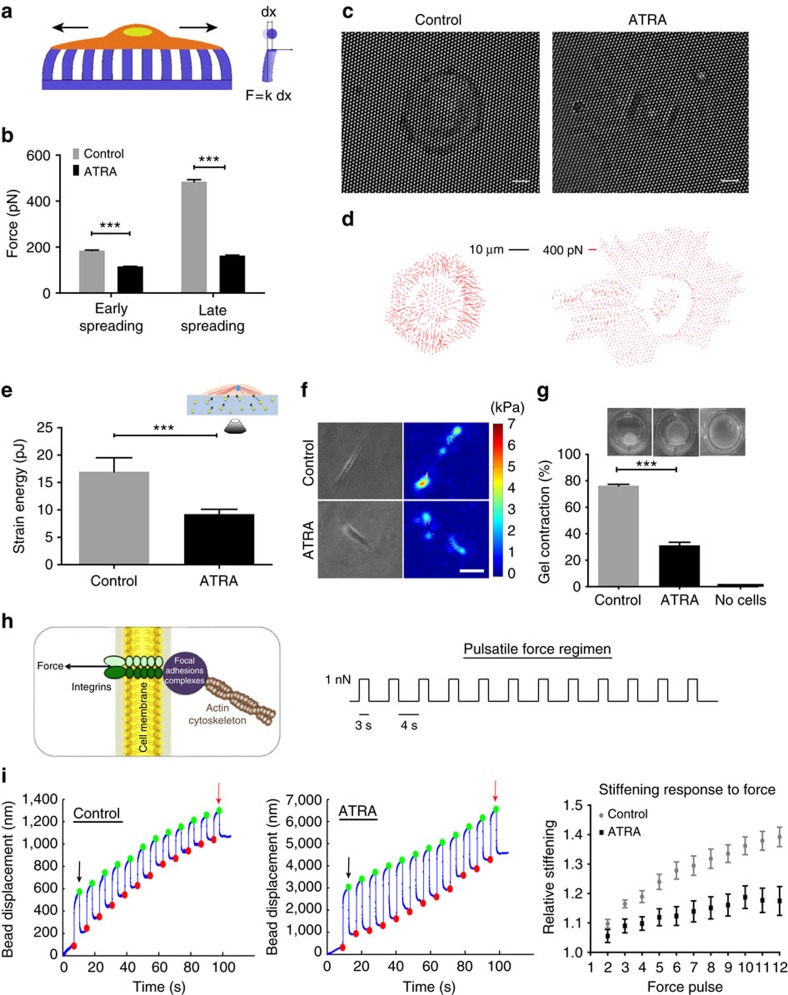
ATRA reduces traction forces and impairs mechanosensing capacity in PSCs. (**a**) Schematic representation of the elastic pillars microsensors (*F*, force; *x*, distance). (**b**) Average force applied on each pillar for early and late spreading. *n*>10 in all cases. (**c**) Bright-field images of representative PSCs on top of the micropillars—early spreading phase. Scale bar, 10 μm. (**d**) The respective force vector maps indicating the magnitude and direction of applied forces calculated from the maximum pillar displacement through a custom-built tracking algorithm. (**e**) Strain energy (mean±s.e.m., *n*>300 cells per condition) imparted by stationary PSCs. (**f**) Bright-field images of representative PSCs on FN-coated, polyacrylamide gels and corresponding traction maps. (**g**) Images show gel contraction by PSCs. Histogram shows quantification of *n*>10 gels per condition assessed over multiple experiments. (**h**) Left, diagram of the cell–matrix interface showing application of mechanical tension onto integrin receptors to mimic the transmission of mechanical force from the extracellular matrix to the cytoskeleton via the mechanical bridge formed by integrins and focal adhesions. Right, pulsatile force regimen applied with magnetic tweezers. (**i**) Left and middle, representative traces tracking the displacement of the beads in response to the force (mechanosensing). The amplitude of the bead displacement was progressively reduced in control PSCs, showing that cells detected force application and responded by stiffening their cytoskeleton. In ATRA-treated PSCs, the reduction in bead displacement was severely suppressed indicating impaired ability to detect external mechanical stimuli. Black and red arrows indicate initial and final amplitude of the bead oscillation, respectively. Right, cell stiffening response to force, (*P*<0.001, *n*=26 traces for control, *n*=34 for ATRA). When not specified, results are expressed as mean±s.e.m. In all panels, error bars represent s.e.m. *P<0.05; **P<0.01; ***P<0.001; (*t*-test); three or more experimental replicates.

Transition of quiescent PSCs to an activated myofibroblastic phenotype is marked by profound cytoskeletal changes and elevated actomyosin contractility^4^,^26^. Indeed, acquisition of cytoskeletal contractility is required for the emergence, maintenance and functional activity of CAFs^13^. While transient activation of PSCs is adaptive in the context of a wound-healing scenario, the unabated and persistent activity of the myofibroblastic phenotype in PDAC represents a mal-adaptive response that can exacerbate desmoplasia and lead to metastasis^27^. ATRA is an active vitamin-A metabolite with pleiotropic action and potential to induce genome-wide transcriptional changes to influence PSC activation. To investigate whether ATRA treatment leads to the changes in the pattern of forces that PSCs exert on their substrate, we seeded cells on top of a substrate consisting of an array of elastic polydimethylsiloxane micropillars coated with FN. The deflection of each pillar is optically monitored and is proportional to the traction force (Fig. 2a). Untreated control PSCs generated elevated traction stresses that were mainly concentrated around the cell periphery (Fig. 2c,d; Supplementary Fig. 2; Supplementary Movie 1). ATRA-treated PSCs by contrast showed a marked decrease in the overall traction force generation during the early and late stages of the spreading phase (Fig. 2b).

To explore whether the difference in traction force generation was also present under steady-state conditions following the spreading phase, we seeded untreated control or ATRA-treated PSCs on FN-coated polyacrylamide gels with embedded fluorescent microbeads that served as fiducial markers of traction-induced gel deformations. Before and after cell trypsinization, fluorescent images were taken to measure the displacement field of the gel and derive the cell traction stresses and the associated elastic strain energy of the deformed gel. ATRA-treated PSCs displayed a reduced ability to deform the underlying matrix, with a twofold decrease on the strain energy of the matrix, while displaying significantly larger FAs compared with control cells (Fig. 2e,f; Supplementary Fig. 3). Similarly, contraction of a matrigel-collagen gel seeded on top with PSCs showed the same trend, whereby ATRA-treated PSCs had a severely reduced ability to deform the collagen matrigel matrix after 65 h, thereby confirming that ATRA treatment inhibits force generation in PSCs (Fig. 2g).

The higher capacity of cells to apply forces on their substrates also correlates with increased cytoskeletal stiffness^28^,^29^. To examine the cytoskeletal stiffness, we used magnetic tweezers microrheology (Supplementary Fig. 4). All cells exhibited a viscoelastic response consistent with power-law rheology. In agreement with our previous observations, ATRA-treated PSCs displayed a threefold increase in compliance (reciprocal of stiffness) compared with untreated control cells, suggesting a decrease in overall cytoskeletal tension. By contrast, differences in cell compliance between control and ATRA-treated cells were largely offset when PSCs were pretreated with blebbistatin, an inhibitor of myosin II ATPase activity, confirming that ATRA induces cell softening mainly through a reduction of actomyosin contraction. These results collectively demonstrate that ATRA suppresses traction force generation and reduces cytoskeletal stiffness of PSCs in an actomyosin-dependent manner.

### ATRA treatment suppresses PSC mechanosensing

The ability to sense and respond to exogenous mechanical forces, also referred as mechanosensing, is a key determinant of tissue homoeostasis, tumour progression and phenotypic maintenance of tumour myofibroblasts^13^,^21^,^22^. To investigate whether ATRA affects mechanosensing in PSCs, we utilized magnetic tweezers to apply controlled forces on integrins through magnetic beads coated with FN and measured the bead displacement during successive pulses of constant force. Localized force application on integrins triggers an adaptive cellular stiffening response resulting in decreased bead displacement, also called ‘reinforcement'. This adaptive stiffening response involves remodelling of the actin cytoskeleton and requires the RhoA–ROCK–MLC-2 signalling axis^18^. Cytoskeletal stiffening in response to force presumably represents an adaptation that allows the cell to modulate its own mechanically active biochemical network within a mechanical feedback loop. While untreated control PSCs displayed robust reinforcement and intact mechanosensing (39% increase in relative cellular stiffness at the end of the pulsatile force regime), ATRA treatment significantly suppressed reinforcement (only 17% increase in relative cellular stiffness) and, thus, the ability of PSCs to respond to extracellular mechanical cues.

After confirming quantitatively that ATRA reduces the PSCs ability to generate forces on the ECM and to sense external mechanical stimuli, the tandem that perpetuates the activating mechanical loop in myofibroblasts, we tested the levels of PSC activation. Alpha smooth muscle actin and vimentin, two widely used markers for myofibroblasts activation, were significantly reduced in ATRA-treated PSCs, while desmin, a marker for PSC quiescence, was markedly increased (Supplementary Fig. 5). Collectively, these results show that ATRA suppresses the ability of PSCs to sense extracellular mechanical cues (mechanosensing) and induces cytoskeletal changes consistent with a quiescent-like phenotype.

Fibroblasts and stellate cells are highly mechanosensitive cells that respond to stiff microenvironments by differentiating towards a myofibroblastic phenotype, even in the absence of soluble profibrotic factors, thus establishing a vicious cycle of increased fibroblast activation, increased matrix stiffness and fibrosis^14^,^30^. Inhibiting mechanosensory-driven activation of myofibroblasts has been proposed as a novel therapeutic strategy to target myofibroblast activation and fibrosis^14^. Targeting common mechanotransduction pathways involving Rho-mediated actomyosin contraction, integrin-based focal adhesions or mechanosensitive transcription factors could all represent attractive targets for this purpose.

### ATRA inhibits matrix remodelling and cancer cell invasion

Although the tumour-promoting features of activated PSCs have been traditionally ascribed to paracrine signalling via growth factors and cytokines^3^,^31^, recalcitrant activation of PSCs perturbs not only the biochemical but also the biomechanical homoeostasis of the tumour microenvironment^13^,^21^,^22^. Force-dependent tissue remodelling and ECM stiffening can create a permissive microenvironment for local cancer cell invasion via modification of the mechanoreciprocal tumour stroma crosstalk^13^,^21^,^32^,^33^,^34^. The ability of fibroblasts to contract collagen gels correlates with their ECM remodelling capacity^13^.

**Figure 3:**
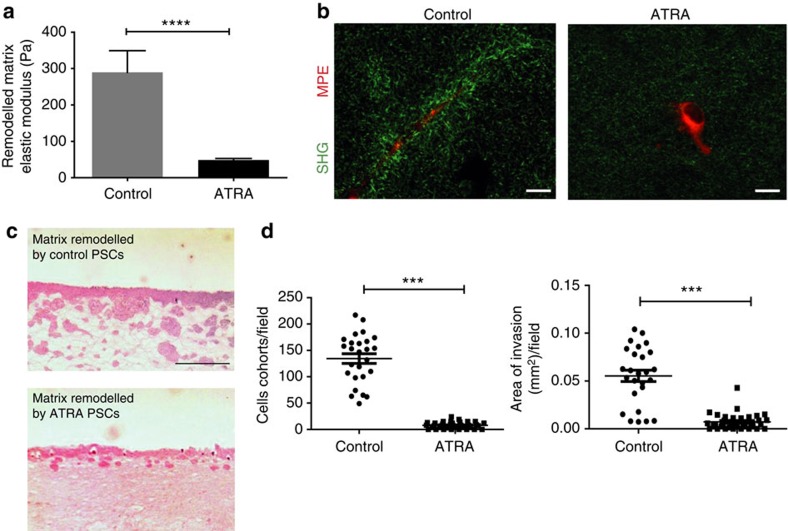
ATRA impairs PSCs capacity to remodel the ECM to promote cancer invasion. (**a**) Elastic modulus for collagen Matrigel matrices previously remodelled by PSCs expressed as mean±s.e.m.; control *n*=113 and ATRA *n*=114 measurements obtained in three independent experiments. (**b**) Collagen second-harmonic signals (green) and cells (red). Scale bar, 20 μm. (**c**) Representative images of haematoxylin and eosin immunofluorescence staining showing AsPC1 cancer cell invasions. Scale bar, 50 μm. (**d**) AsPC1 invasion expressed as number of invading particles and area, respectively. Each point represents a different section. In all panels, ***P<0.001; ****P<0.0001; (*t*-test).

We employed 3D organotypic assays to investigate the effect of ATRA on the PSC capacity to remodel the matrix to promote cancer cell invasion. Using atomic force microscopy (AFM) and second harmonic generation (SHG) imaging, we tested the stiffness and collagen fibre organization of matrices previously embedded with and remodelled by untreated control or ATRA-treated PSCs. While untreated PSCs were able to stiffen the matrix substantially, ATRA treatment suppressed the remodelling ability of PSCs and decreased matrix stiffness by fivefold, as measured through the elastic modulus of the gels; furthermore, remodelled matrices by untreated PSCs were also associated with thicker bundles of collagen fibrils, not observed in matrices remodelled by ATRA-treated PSCs (Fig. 3a,b).

To investigate whether ATRA additionally interferes with proteolytic remodelling of PSCs, we assayed the levels of matrix metalloproteinases (MMPs). MMPs are a family of calcium-dependent zinc containing proteases that hold central roles in a wide range of physiological and pathological processes, and have the ability to degrade proteins in the ECM^35^. MMP-2 and MMP-9 are the two more relevant MMPs synthesized by PSCs^36^. While MMP-2 levels in PSCs under ATRA treatment were not significantly different from control PSCs, we observed a significant fivefold increase in MMP-9 levels in ATRA-treated PSCs in comparison with control PSCs (Supplementary Fig. 6). The activities of MMPs are tightly regulated by endogenous tissue inhibitors of metalloproteinases (TIMPs) and the balance between MMPs and TIMPs governs the eventual ECM and tissue remodelling. TIMP-1 is a specific MMP-9 inhibitor^35^,^37^. Our data show a significant 30% reduction in TIMP-1 in ATRA-treated PSCs with respect to control PSCs (Supplementary Fig. 6). Previous studies on the effect of ATRA on MMPs are overlapping and contradictory. While some studies show that ATRA upregulates MMPs^38^, others reported an opposite effect for ATRA in different cell types^39^,^40^,^41^. A possible explanation for this divergence is that ATRA exerts its effect by binding to different isoforms of the RARs, alpha, beta and gamma. The distribution of these isoforms is different depending on the cell type and ATRA might activate this range of isoforms in a different manner in each cell type^42^. More MMP-9 and less TIMP-1 levels might have anti-fibrotic roles, and therefore be beneficial to resolve PSC-associated fibrosis. However, the analysis of the profibrotic and anti-fibrotic roles of MMPs and TIMPs is more complex and requires further experimentation as MMPs and TIMPs have been shown to have both stimulatory and inhibitory roles in fibrosis^43^.

We further interrogated whether PSC-remodelled matrices were rendered permissive for the invasion of pancreatic cancer cells. Untreated control and ATRA-treated PSCs were left to remodel the matrices for 3 days before being killed and washed out from the matrices. The ability of AsPC1 pancreatic cancer cells to invade into these matrices was then evaluated and quantified (Fig. 3c,d). We found that pancreatic cancer cells invaded deeply into matrices previously remodelled by activated control PSCs, but showed minimal capacity to invade matrices remodelled by ATRA-treated PSCs.

These findings support that ATRA reprograms PSCs to suppress force-mediated and proteolytic matrix remodelling and peritumoural tissue stiffness to inhibit subsequent cancer cell invasion. The reduced invasive ability of cancer cells can be attributed to the change in the mechanical and/or topographical characteristics of the remodelled matrices promoted by ATRA treatment on PSCs. Lines of tension and CAF-generated ECM tracks are known to serve as guidance cues for collective cancer invasion^20^ and generally a stiffer matrix promotes invasive phenotypes in malignant epithelium by forcing tumour progression through mechanotransduction^32^.

### ATRA downregulates MLC-2 actomyosin contractility

**Figure 4:**
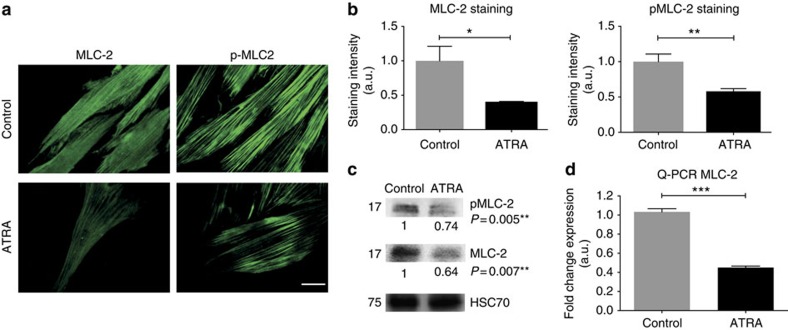
ATRA downregulates MLC-2 actomyosin contractility. (**a**) Immunofluorescent images of total myosin light-chain 2 (MLC-2) and phosphorylated levels (pMLC-2). (**b**) Quantification of staining intensity (*n*>20 cells in all cases and three experimental replicates). Scale bar, 50 μm. (**c**,**d**) MLC-2 level expression at the protein (western blotting) and mRNA (qPCR) levels. All results are expressed as mean±s.e.m. and *P<0.05; **P<0.01; ***P<0.001; (*t*-test), three independent experiments for western blotting and qPCR.

To investigate the signalling pathway targeted by ATRA treatment in PSC, we focused on MLC-2, which controls actomyosin contraction and is causally associated with CAF emergence and maintenance through a feed-forward loop that entails ECM stiffening and elevated integrin mechanosignalling^13^. We observed a marked downregulation in the expression of MLC-2 at the gene and protein levels. Accordingly, the phosphorylated level of MLC-2 was proportionally reduced (Fig. 4a–d; Supplementary Fig. 7). The effect of ATRA treatment on the levels of MLC-2 was confirmed by 3D gel contraction assays, in which MLC-2 rescue in ATRA-treated PSCs recovered their capacity to contract the gel at similar levels to control PSCs (Supplementary Fig. 8).

To test whether the observed PSC response to ATRA treatment may be affected by prior association with cancer cells, we collected media from AsPC1 pancreatic cancer cells, and grew PSCs on 40% AsPC1-conditioning media for 24 h, and then tested the effect of ATRA treatment on the sizes of PSC focal adhesions, the MLC-2 levels and the PSC ability to apply forces on their matrices (Supplementary Fig. 9). In agreement with our previous observations, ATRA treatment significantly increased the size of focal adhesions, significantly reduced MLC-2 levels in PSCs previously exposed to cancer cell conditioning media and markedly decreased their ability to apply forces to contract the 3D matrigel-collagen gels.

**Figure 5:**
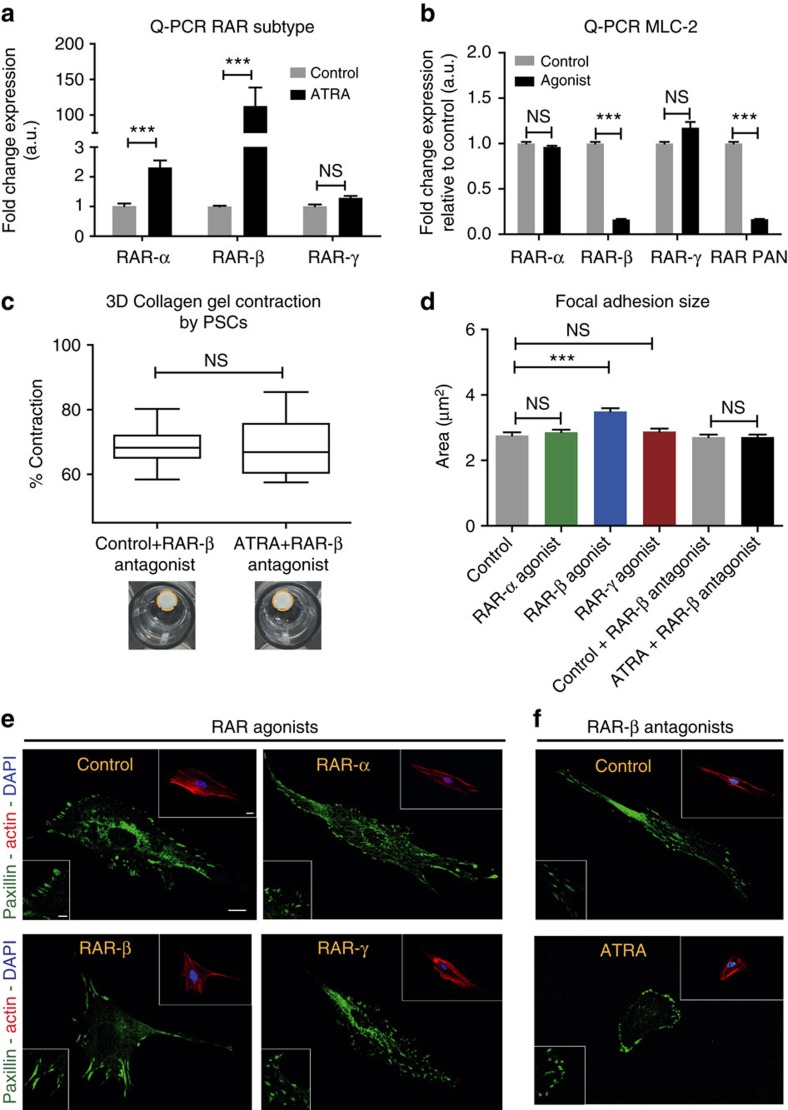
ATRA biomechanically reprograms PSCs in a RAR-β-dependent manner. (**a**) qPCR mRNA levels of the different retinoic acid receptor (RAR) subtypes, RAR-α, RAR-β and RAR-γ in untreated control and ATRA-treated PSCs. Bars represent mean±s.e.m. (**b**) qPCR mRNA levels of MLC-2 in control PSCs and in PSCs treated with agonists for RAR-α, RAR-β and RAR-γ. Bars represent mean±s.e.m. (**c**) 3D gel contraction assay using control and ATRA-treated PSCs in the presence of RAR-β antagonist. In the box-and-whisker plot, the central box represents values from the lower to the upper quartile. The middle line represents the mean. The vertical line extends from the minimum to the maximum value. Control and ATRA 12 and 15 gels, respectively, assessed in three independent experiments. NS, no significant differences. Dotted yellow lines represent the gels contours of representative images of gel contraction. (**d**) Quantification of focal adhesions size of the images presented in **e**,**f**. Bars represent mean±s.e.m. (**e**) Representative images of focal adhesions size in PSCs treated with agonists for RAR-α, RAR-β and RAR-γ. (**f**) Representative images of focal adhesion sizes in PSCs untreated control and ATRA treated in the presence of RAR-β antagonist. Scale bars for **e**,**f**: main image 20 μm; focal adhesion inset 5 μm; and blue/red channels inset 20 μm. In all panels, data were collected during three or more independent experiments and *P<0.05; **P<0.01; ***P<0.001; (*t*-test).

We then sought to study the mechanism that underpins the observed effect of ATRA on PSCs. There are two families of retinoic acid nuclear receptors, RAR or retinoic acid receptor and RXR or retinoid X receptor, which selectively bind the two main forms of retinoic acids, ATRA and 9-RA (9-cis-retinoic acid). ATRA is an exclusive ligand of the three subtypes of RAR (RAR-α, β and γ)^44^,^45^,^46^. PSCs were tested for expression of the different subtypes of RAR (RAR-α, β and γ) at the mRNA level. PSCs abundantly expressed RAR-α and RAR-γ but only minimal levels of RAR-β (Supplementary Fig. 10). ATRA treatment did not affect the expression of RAR-γ, but increased threefold the expression level of RAR-α, and induced a pronounced increase in RAR-β expression of 100-fold (Fig. 5a), which is in agreement with previously reported data^25^.

Next, we used a battery of RAR agonists and antagonists to learn more about which specific RAR subtype modulates the ATRA effect on PSC focal adhesions and MLC-2 levels, which mediates actomyosin-dependent traction forces, and mechanosensing^18^,^19^. Exposing PSCs to 1 μM of the selective RAR-α (AM580)^44^ or selective RAR-γ (BMS961)^47^ agonists did not affect the MLC-2 levels or the focal adhesion sizes in PSCs. However, treating PSCs with 1 μM of the selective RAR-β (cd2314)^46^,^48^ or pan-RAR agonist (TTNPB)^49^,^50^ significantly reduced the MLC-2 levels fivefold, and increased the size of the focal adhesions ∼30% similarly to the observed effect of ATRA on MLC-2 levels and focal adhesion sizes. Furthermore, we observed that the effect of ATRA on the size of focal adhesion and PSC ability to deform 3D matrigel-collagen gels is reverted in the presence of 0.1 μM of the RAR-β antagonist (cd2665)^51^,^52^ (Fig. 5b–e). Further studies are needed to elucidate whether ATRA regulates the transcription of MLC-2 gene directly through a functional retinoic acid response element on its promoter region or indirectly via an intermediary transcription factor. Taken together, these data suggest that MLC-2 is transcriptionally downregulated in PSCs by ATRA in a RAR-β-dependent manner, and implicates a new role for RAR-β signalling in controlling traction force generation, mechanosensing and biomechanical matrix remodelling.

### ATRA induces the formation of dorsal stress fibres

Stress fibres are made of crosslinked F-actin filament bundles that are distinctly characterized by their functions, cellular localization and composition. Ventral stress fibres are myosin rich fibres that form at the ventral surface of spreading and migrating cells and generate strong traction forces^53^. In addition, the lamellar region of adherent cells contains radial or dorsal stress fibres, which perpendicularly elongate from the leading edge. These dorsal stress fibres assemble in a myosin-independent manner, are non-contractile, and have primarily a structural role serving as a template for the maturation of integrin-based focal adhesions^54^. The fact that ATRA transcriptionally downregulates MLC-2 levels and concurrently increases the size of focal adhesions in PSCs prompted us to characterize the abundance of ventral and dorsal fibres in control and ATRA-treated PSCs as a potential mechanism to reconcile the discrepancy. Interestingly, we observed a significant threefold increase in the ratio of dorsal/ventral fibres in ATRA-treated PSCs in comparison with control PSCs (Supplementary Fig. 11a,b).

Dorsal stress fibres are known to assemble downstream of active Rac1, but not RhoA. After we observed that dorsal stress fibres predominate in ATRA-treated PSCs, we tested the levels of activation of both RhoA and Rac1 in untreated control and ATRA-treated PSCs. While we saw no significant differences in the levels of active RhoA, we observed a significant 20% increase in the activation of Rac1 in ATRA-treated PSCs when compared with control PSCs (Supplementary Fig. 11c). Dorsal stress fibres are known to promote early cell spreading through Rac-1-induced actin polymerization and membrane extension in newly spreading cells^54^. The increased levels of active Rac1 in ATRA-treated PSCs might provide an explanation for the higher spreading rate of PSCs with ATRA treatment. Assembly of dorsal stress fibres in the lamellar cytoskeleton, while not necessary for force transmission or indicative of the overall force-generating potential of a cell, can nevertheless provide a stable scaffold for the recruitment and clustering of focal adhesion proteins even when actomyosin tension is suppressed by as much as 80% (ref. 55). Taken together, the increased assembly of non-contractile dorsal stress fibres in ATRA-treated PSCs might explain the presence of larger focal adhesions despite a reduction in actomyosin contractility and force transmission to the ECM.

### ATRA impedes PSC migration

CAFs are known to participate in the formation of distant metastatic sites by co-migrating with tumour cells by first remodelling the matrix ahead of them leaving ‘tracks' in the matrix for the invading carcinoma cells to follow^20^. It is also well documented that stellate cells can infiltrate the stroma and migrate towards the tumour to communicate with the cancer cells in response to chemokine activation^56^,^57^,^58^. Therefore, suppressing the migration ability of stellate cells may have important roles in slowing down the formation of metastatic niches and abrogating the crosstalk between PSCs and cancer cells. Using a scratch would-healing assay, we found that ATRA treatment severely inhibits the migratory activity of PSCs (Supplementary Fig. 12). This result cannot necessarily be attributed to reduced traction forces in PSCs, as previous studies have shown no association between traction forces and cell migration speed^59^, which is in line with other reports indicating that blebbistatin inhibits contraction but paradoxically accelerates migration in mouse embryonic stem cells^60^ and hepatic stellate cells^61^.

The impaired migration in ATRA-treated PSCs could be explained by other mechanisms. Cell migration is a tightly regulated process dependent on dynamically coordinated adhesion formation and turnover at the cell front along with adhesion disassembly and tail retraction at the cell rear. The increased size of focal adhesions and cell–matrix adhesive capacity in ATRA-treated cells might physically impede migration by providing a stronger attachment to the underlying substrate, thereby interfering with normal spatiotemporal focal adhesion dynamics and preventing efficient cell movement. Second, wounding-induced PSC migration relies on chemotactic factors such as PDGF, which can be produced in an autocrine manner by activated PSCs^26^. Recent reports have shown that ATRA downregulates PDGFRβ at the mRNA level in activated PSCs^62^ and this could represent another possible mechanism for disrupting wounding-induced migration in PSCs.

## Discussion

**Figure 6:**
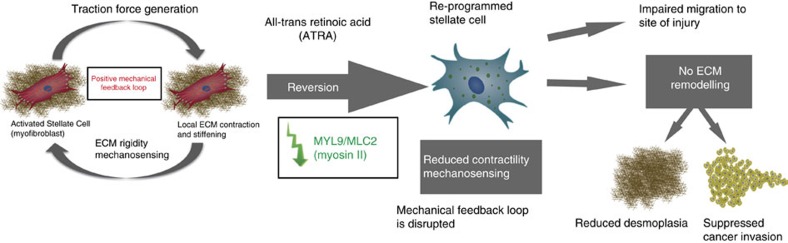
Model for the biomechanical reprogramming of PSCs. ATRA downregulates myosin II-dependent force generation and mechanosensing in PSCs. PSCs are reprogrammed to a more quiescent phenotype, cannot migrate, remodel the ECM or promote cancer invasion.

Retinoids are vitamin A derivatives with a long history as chemotherapeutic agents in oncology due to their cell differentiative properties. PDAC is an aggressive malignancy that features disrupted retinoid signalling and particularly low expression and activity of RARs that correlates with poor patient survival^63^. The pleiotropic action of retinoids makes them good candidates as stromal-reprogramming agents for tumours exhibiting pronounced desmoplasia such as pancreatic cancer. Here we found that human PSCs express all three RARs, albeit at different levels, and that treatment with ATRA, the physiologically active metabolite of vitamin A, mechanically reprograms PSC to promote quiescence *in vitro* and inhibit pancreatic cancer cell invasion. We uncover a previously unidentified mechanism through which activation of the RAR-β negatively regulates transcription of MLC-2 in PSCs. This activation suppresses actomyosin contraction, mechanosensing and force-mediated matrix remodelling and stiffening, which, in turn, creates a microenvironment unfavourable for invasion by pancreatic cancer cells. The reduced invasive behaviour of cancer cells was independent of paracrine signalling between PSCs and cancer cells, and was mostly attributed to the altered biomechanical and/or topographical characteristics of the remodelled microenvironment promoted by ATRA treatment on PSCs (Fig. 6).

This study was conducted using human culture-activated PSCs—a widely used *in vitro* model that recapitulates with good approximation the *in vivo* activated phenotype that occurs in cancer or persistent injury. However, the activated PSC phenotype resulting from culture-induced transdifferentiation can differ, to some degree, from the cancer-associated phenotype activated through exposure to the tumour microenvironment. Although exposure of PSCs to cancer cell supernatant did not change the overall PSC response to ATRA in our study, this approach cannot fully represent cancer-associated PSCs, because *in vivo*, the latter would be affected not only by cancer cells, which are themselves heterogeneous, but also by other cells in the stroma. Thus, future studies utilizing cancer-associated PSC is warranted for further validation.

High levels of actomyosin (MLC-2)-mediated contractility is an indispensable feature for the emergence and functional persistence of tumour-associated myofibroblasts in the stroma; and MLC-2 is found to be consistently elevated in CAFs as opposed to normal fibroblasts^13^. Although paracrine factors released from the tumour are important for the initial activation of PSCs^26^, this self-sustaining behaviour is biomechanically regulated through a feed-forward loop involving actomyosin-mediated ECM stiffening and stiffness-sensing leading to PSC activation^13^,^21^,^64^.

We found that ATRA lowers the magnitude of traction forces PSCs exert on their substrate and suppresses their ability to respond to external mechanical cues from the ECM, which may collectively interrupt the mechanosensory-driven activation of PSCs. In accordance with this, we found that ATRA induced the expression of cytoskeletal markers associated with PSC quiescence such as a reduction in Alpha smooth muscle actin and vimentin, and an increase in desmin. Interestingly, previous reports have shown that PSC activation could be blocked by ROCK inhibitors Y-27632 and HA-1077 (fausidil)^65^. We note that MLC-2 may serve as a convergence point for multiple environmental factors to regulate myofibroblastic differentiation of PSCs. For example, integrin-mediated mechanotransduction, mechanical stretch and contractile agonists, such as endothelin-1 and angiotensin II, are known to regulate smooth-muscle-specific gene expression as well as the MLC-2 axis^65^.

It is worth noting that two independent investigations previously reported that retinoic acid inhibit PSC activation via suppressing the Wnt/β–catenin signalling pathway to either suppress pancreatic fibrosis in a mouse model of chronic pancreatitis or slow pancreatic tumour progression^25^,^62^. We report an additional mechanism by which ATRA mechanically reprograms PSC via transcriptional repression of MLC-2 to normalize matrix remodelling and inhibit cancer invasion in the absence of any secreted paracrine signalling. It is currently unknown if MLC-2-dependent actomyosin activity could be linked to the Wnt/β-catenin pathway; however, there is a literature report from Samuel *et al.* implicating actomyosin-mediated cellular contractile force with tissue stiffness and increased activation of β-catenin (a known mechanically activated transcription factor) to force tumour progression in a mouse model of skin cancer^66^. We suggest that RAR-β-selective agonists could be used to mechanically reprogram the stroma by targeting the actomyosin function of persistently activated PSC.

We also found that ATRA reprogrammed cell-to-ECM adhesion in PSCs resulting in stronger ECM adhesion and increased focal adhesion (talin and paxillin) size. Focal adhesions behave as ‘molecular clutches' linking the ECM to intracellular cytoskeleton and allowing bidirectional transmission of force across the plasma membrane. The notion that focal adhesion maturation is regulated by actomyosin contractility has been called into question by recent reports that suggest that focal adhesion size is a poor predictor of the overall tension state of the cell^67^,^68^,^69^ and our findings also point to this direction. Our observation that ATRA induces secondary cytoskeletal changes such as the formation of Rac1-dependent non-contractile dorsal stress fibres could explain the higher rates of cell spreading in ATRA-treated PSCs. The increased presence of myosin II-independent dorsal stress fibres in ATRA-treated PSCs that serve as structural templates for increased focal adhesion growth^55^ (in a myosin-independent manner) could reconcile the inverse relationship between focal adhesion size and traction forces in ATRA-treated cells.

The increasing appreciation that mechanotransduction and cell mechanical properties are key aspects in stromal biology and maintenance of the CAF phenotype in solid tumours has recently led to the discovery of YAP^13^ and caveolin-1 (ref. 21) as key mechanoregulators of CAF function and biomechnical homoeostasis of the stroma with implications for metastatic progression. Our findings expand the aforementioned list by highlighting RAR-β as a genomic regulator of actomyosin tension, mechanosensing and microenvironmental remodelling in PSCs.

Ablation of the desmoplastic stroma or genetic deletion of activated myofiboblasts have met with limited success in the realm of stroma-targeting approaches for the treatment of PDAC^6^,^10^. Due to the multi-faceted role of the desmoplastic stroma in both promoting and restraining PDAC progression^9^, stromal-reprogramming strategies that aim to reprogram PSCs to a quiescent-like state are highly sought after. Recently, the vitamin D receptor has shown promise in this direction. We herein suggest that ATRA or selective RAR-β agonists with improved toxicity profile, or potentially compounds that block actomyosin tension, can open new avenues in the treatment of PDAC by biomechanically reprograming PSCs to a quiescent-like state, and restoring the biomechanical homoeostasis of the microenvironment to inhibit cancer invasion and progression.

## Methods

### Cell culture and antibodies

Primary, culture-activated human PSCs (passages 6–8, HPaSteC #3830—Caltag Medsystems, UK) were exposed to ATRA dissolved in ethanol at a concentration of 1 μM for 10 days. Cell viability data are shown in Supplementary Fig. 13. Medium was changed every 24 h and the drug treatment was performed in subdued light. Cells incubated with culture medium (DMEM with 2% fetal bovine serum (FBS), 1% penicillin/streptomycin and 1% fungizone antimycotic) with an equivalent amount of vehicle (0.1% ethanol) served as controls. Antibodies: HSC70—Santa Cruz (1/10,000), Talin—Abcam ab71333 (1/100), Paxillin—BD Biosciences 610051 (1/100), MLC-2—Millipore MABT180 (WB 1/100, IF 1/200), pMLC-2 (Thr18/Ser19)—Cell Signalling, 3674 (WB 1/100, IF 1/200), Anti-Mouse HRP—Invitrogen 626580 (1/2,000), Anti-Rabbit HRP—Abcam ab137914 (1/2,000) and Anti-Mouse 488—Invitrogen-A11029 (1/400). RAR-α agonist (AM580), Tocris 0760; RAR-β agonist (cd2314), Tocris 3824; RAR-γ agonist (BMS 961), Tocris 3410; pan-RAR agonist (TTNPB), Tocris 0761. In all cases, PSCs were exposed to 1 μM of agonist during 24 h. RAR-β antagonist (cd2665), Tocris 3800. For the experiments using the AsPC1-conditioning media, cells were grown under normal culture media with 10% FBS until 80% confluency, washed three times with PBS and grown in serum-free media for 24 h. The medium was collected and use in a proportion 40% conditioned media and 60% PSC media to grow PSCs for 24 h.

### Western blotting

The cell lysates were prepared with radio immunoprecipitation assay buffer (Sigma, R0278) containing proteinase inhibitors (Sigma, P4340). The protein concentration was quantified by DC protein assay (Bio-Rad, 500-0113) according to the manufacturer's instructions. Samples were separated by an SDS–polyacrylamide gel electrophoresis gel under reducing conditions and transferred to a nitrocellulose membrane (GE Healthcare, 10401196), and then blocked with 5% bovine serum albumin (BSA; Sigma, A8022)–0.1% Tween-20 (Sigma, P1379) in PBS. All primary antibodies were prepared in blocking solution and incubated overnight at 4 °C. The membrane was washed and incubated with horseradish peroxidase (HRP)-conjugated secondary antibodies in blocking solution for 1 h at room temperature. Finally, the membrane was washed and developed with HRP substrate (Millipore, WBLUR0100; Supplementary Fig. 14).

### RT–PCR

Total RNA was extracted using the RNeasy Mini kit (Qiagen, 74104) and 1 μg of total RNA was reverse-transcribed using the High-Capacity RNA-to-cDNA kit (Applied Biosystems, 4387406) according to the manufacturer's instructions. QPCR was performed using the SYBR Green PCR Master Mix (Applied Biosystems, 4309155) with 100 ng cDNA input in 20 μl reaction volume. GAPDH expression level was used for normalization as a housekeeping gene. The primer sequences for MLC-2 were as follow: forward, 5′-ATCCACCTCCATCTTCTT-3′ and reverse, 5′-AATACACGACCTCCTGTT-3′. The sequences were as following: MMP-2: forward-5′-TCTCCTGACATTGACCTTGGC-3′, reverse-5′-CAAGGTGCTGGCTGAGTAGATC-3′; MMP-9: forward-5′-TTGACAGCGACAAGAAGTGG-3′, reverse-5′-GCCATTCACGTCGTCCTTAT-3′; TIMP-1: forward-5′-TCAACCAGACCACCTTATACCA-3′, reverse-5′-ATCCGCAGACACTCCAT-3′; MLC-2: forward, 5′-ATCCACCTCCATCTTCTT-3′ and reverse, 5′-AATACACGACCTCCTGTT-3′; GAPDH: forward-5′-ACAGTTGCCATGTAGACC-3′, reverse-5′-TTTTTGGTTGAGCACAGG-3′. All primers were used at 300 nM final concentration. The relative gene expression was analysed by comparative 2^−ΔΔct^ method.

### Immunofluorescence staining

All immunofluorescence staining was done on coverslips coated with 10 μg ml^−1^ FN (Sigma, F0895). Following pertinent treatment cells were fixed with 4% paraformaldehyde (Sigma, P6148) in PBS for 10 min, and then blocked and permeabilized with 0.2% BSA–0.1%Triton (Sigma, T8787) in PBS for 30 min. After blocking, cells were incubated with primary antibodies prepared in blocking solution for 1 h at room temperature in a humidified chamber. Then, cells were washed in PBS and incubated with Alexa Fluor 488-conjugated secondary antibodies and Phalloidin (Invitrogen, A22283, 1/1,000 dilution) prepared in PBS for 30 min at room temperature. Finally, coverslips were washed in PBS and mounted in mounting reagent with 4,6-diamidino-2-phenylindole (Invitrogen, P36931). The method for the immunostaining quantification is provided in Supplementary Information.

### Measurement of rate of cell spreading and spread area

Measurements of the time-dependent spreading of cells were conducted on glass bottom Petri dishes (Maktek) coated with human plasma FN (10 μg ml^−1^; Sigma) and incubated at 37 °C. Cells were trypsinised before measurements, suspended in culture medium (DMEM with 2% FBS) and plated on the dishes. Images of the cells were obtained with an inverted microscope (Eclipse Ti; Nikon) in DIC mode with the samples held at 37 °C. Images were obtained with a sCMOS camera every 5 min using a × 20 (0.4 numerical aperture (NA), air; Nikon) objective until noticeable cell spreading had stopped. The cell area was calculated using the imaging software (NIS elements; Nikon) by selecting the perimeter of the cell in each frame allowing the cell area to be tracked with time.

### Micropillar video microscopy and traction force measurements

Pillar arrays were coated with human plasma FN (10 μg ml^−1^; Sigma) and incubated at 37 °C for 1 h before measurements. Cells that had been trypsinised before measurements were suspended in culture media and plated onto the pillar substrates. Time-lapse imaging of the pillars was conducted with an inverted microscope (Eclipse Ti; Nikon) operating in bright-field mode with the samples held at an ambient temperature of 37 °C. Image sequences were recorded with a sCMOS camera (Neo sCMOS Andor) at 0.5 Hz using a × 40 (0.6 NA, air; Nikon) objective over the early spreading phase (*t*<60 min) and late spreading phase (90 min<*t*<120 min). The position of each pillar in the time-lapse videos was tracked using a custom MATLAB program to track the centre of a point spread function of the intensity of the pillars across all frames. By selecting a location free of cells, tracking of a small set of pillars allowed a measurement of the stage drift to be obtained and corrected for in the data set. The time-dependent displacement of a given pillar was obtained by subtracting the initial position of the pillar (zero force) from the position in a given frame. Traction forces were obtained by multiplying the pillar displacements by the pillar stiffness, the maxima for each pillar were found to obtain the peak forces across the cell. More information about the pillar fabrication can be found in Supplementary Methods.

### Traction force microscopy

Acrylamide (6.8%)–bis-acrylamide (0.36%) gels were casted on non-electrostatic silane-coated glass slides. The Young's modulus of the polyacrylamide gels was 12.8±0.8 kPa. Yellow-green fluorescent 0.5 μm carboxylated beads (Invitrogen) were embedded in the gels and centrifuged (300 *g*) towards the gel surface during polymerization at 4 °C. The beads served as markers for gel deformations. The gel surface was activated with sulfo-SANPAH (Pierce, Bonn, Germany) and coated with 50 μg ml^−1^ FN. After cells adhered to the gels, cell tractions were computed from the gel surface displacements measured before and after force relaxation and detachment of cells (12 h after seeding) with 8 μM cytochalasin-D and 15 μM ML-7 in trypsin/EDTA. Gel deformations were measured using a Fourier-based difference-with-interpolation image analysis^70^. The experiments were performed at 37 °C, 5% CO_2_ and 95% humidity in DMEM containing 10% FCS in a microscope stage incubation chamber.

### Cell–ECM adhesion strength

To evaluate the strength of cell–matrix adhesion of PSCs (control or ATRA treated), 2.8 μm carboxylated magnetic beads (Dynabeads, Life Technologies) were coated with low concentration FN (1:100, FN:BSA) to allow for easier detachment. The beads were added to the cells and incubated for 5–10 min. A pulsatile pulling force regime of a magnitude *F*=1.5 nN magnitude, frequency 1 Hz and duration 60 s was used in an attempt to detach FN beads from the cells (*n*=170 beads from ≥100 cells per condition). ECM adhesion strength was expression as the percentage of detached beads by the end of force application.

### Cell mechanosensing

To assess how PSCs (control or ATRA treated) sense and respond to applied forces emanating from the ECM, 4.5 μm FN-coated magnetic beads coated were subjected to a pulsatile force regimen applied with magnetic tweezers, consisting of a 3 s, 1 nN pulse of force, followed by a 4 s period of rest, repeated for 12 total pulses over a 100 s time course. The ability of the cells to sense and respond to the applied tension was examined from the rapid cell stiffening response evident by the progressive decrease in amplitude of the bead movement (*n*=26 for PSC control and *n*=34 PSC ATRA). More information about the magnetic tweezers and cellular microrheology experiments can be found in Supplementary Methods.

### Two-dimensional collagen gel contraction assay

Initially, wells in 24-well culture plates were pretreated with PBS containing 2% BSA for 1 h at 37 °C, washed twice with PBS and air dried. Ten parts of rat tail collagen I (9.06 mg ml^−1^, Corning) were added to one part of 10 × DMEM, one part of 10 × PBS and 1.25 parts of deionized water (resulting in a final collagen concentration of 4.5 mg ml^−1^), pH adjusted to 7.4 by adding 0.1 M NaOH and 500 μl of this mixture added to each well. Collagen gels were allowed to solidify at 37 °C for 90 min. After gelation, 1.5 × 10^5^ PSCs (control or pretreated with ATRA for 10 days) were layered on top of formed collagen matrices and fed with DMEM, 2% FBS with or without 1 μM ATRA. Collagen gel contraction was evaluated after 60 h by digital image analysis software and expressed as the % reduction in the surface area of the gel.

### 3D ECM remodelling assay

To analyse the ECM remodelling ability of PSCs (control or ATRA treated), Collagen-I (BD Bioscences, 354249, stock concentration 9.37 mg ml^−1^) and Matrigel (BD Bioscences, 354234, stock concentration 9 mg ml^−1^) mixture gels were prepared with one part 10 × DMEM (Sigma, D2429) and one part FBS (Gibco, 10500), yielding to a final concentration of 4.5 mg ml^−1^ Collagen-I and 2 mg ml^−1^ Matrigel. The gel mixture was neutralized with 1 M NaOH (Sigma, S8045), then 5 × 10^5^ cells were embedded in gels in culture media. A measure of 80 μl gel volume was added per well of a 96-well plate, which was pretreated with 2% BSA (Sigma, A8022) for 1 h, washed with PBS and air dried for 10 min. Gels were set 1 h at 37 °C, and then incubated with culture media for 3 days at 37 °C. For SHG microscopy, gels were prepared as explained above. After 3 days of incubation at 37 °C, gels were fixed with 4% paraformaldehyde (Sigma, P6148) in PBS for 1 h at 37 °C, and then washed with PBS and permeabilised with 0.3%Triton X-100 (Sigma, T8787) in PBS for 30 min. After that, gels were blocked with 1% BSA–0.1% Triton X-100 in PBS for 1 h. Gels were washed with PBS and stained with Alexa Fluor 546-conjugated Phalloidin at 1/300 dilution in 1% BSA in PBS for 30 min. Finally, gels were washed two times with PBS.

### Atomic force microscopy

For AFM study, collagen Matrigels were lifted from the 96-well plates before measurement and immediately attached to a Petri dish with a droplet of cyanoacrylate adhesive, applied with a 10 μl pipette tip. After Matrigel attachment (1–2 min), the sample was immersed in culture medium (DMEM with 2% FBS) in order for the AFM measurements to be conducted within a 2 h time period. Measurements of the Matrigels have been conducted on a JPK Nanowizard-1 (JPK Instruments) operating in force spectroscopy mode, mounted on an inverted optical microscope (IX-81; Olympus). AFM pyramidal cantilevers (MLCT; Bruker) with a spring constant of 0.07 N m^−1^ were used with a 35 μm glass bead attached to cantilever tip. Before measurements with the adapted cantilevers, their sensitivity was calculated by measuring the slope of force-distance curve in the AFM software on an empty region of the Petri dish. For indentation tests on the sample, the cantilever was aligned over regions in the middle of the samples using the optical microscope. For each sample, 30 force curves were acquired across six different 100 μm regions, this arrangement allowed force curves to be acquired in locations at least 50–100 μm apart. Force-curve acquisition was carried out with an approach speed of 5 μm s^−1^ and a maximum set force of 1.5 nN. Elastic moduli were calculated from the force-distance curves by fitting the contact region of the approach curve with the Hertz contact model, using the AFM software.

### Multiphoton confocal microscopy

Collagen Matrigel samples were prepared for analysis on Petri dishes via the same method mentioned previously for AFM analysis. All SHG images were obtained using a custom-built multiphoton microscope incorporating an upright confocal microscope (SP5, Leica) and a mode-locked Ti:Sapphire Laser (Mai Tai, Newport Spectra-Physics). Images of the SHG signal from collagen I were collected using an 820 nm excitation with SHG signal obtained with a 414/46 nm bandpass filter and multiphoton autofluorescence signal obtained with a 525/40 nm bandpass filter. A × 25, 0.95 NA water-immersion objective (Leica) was used to deliver excitation signal and to collect the SHG emission signal from the sample. Images with a 200 × 200 μm field of view were obtained with 2,048 pixel resolution and a line rate of 10 Hz giving a pixel resolution of ∼0.1 μm with 3 × averaging on each acquisition to reduce the effect of noise.

### Invasion assays

To assess the effect of ATRA treatment on PSC-driven ECM remodelling-related cancer cell invasion 3D organotypic cultures were employed. Organotypic gels were prepared with 52.5% Rat tail Collagen-I (BD Biosciences, 354236), 17.5% Matrigel (BD Bioscences, 354234), 10% FBS (Gibco, 10500) and 10% 10 × DMEM (Sigma, D2429). Gel mixture was neutralized by adding 1 M NaOH (Sigma, S8045), and then 5 × 10^5^ cells were embedded in gels in pertinent media (10% of total gel volume). A measure of 1 ml gel mixture was aliquoted per well of a 24-well plate. Gels were set at 1 h at 37 °C, and then maintained with the pertinent media for 3 days (when contraction is observed). PSCs were killed by incubating the gels with 400 μg ml^−1^ Hygromycin (Life Technologies, 10687-010) containing culture media for 48 h. After that gels were washed with PBS for 45 min three times. Then, 2.5 × 10^5^ AsPc1 cells (2:1 ratio for PSC: Cancer cell) were seeded on top of the gels and incubated overnight. Gels were lifted to an air liquid interface on top of Rat tail Collagen-I-coated nylon membranes (100 μm pore size, Millipore, NY1H02500) placed on stainless steel grids and fed from beneath for 10 days with 10% FBS containing RPMI (Sigma, R8758). Then, gels were collected, fixed overnight with formalin (Sigma, HT501128-4L) and embedded in paraffin (Fisher, 12624077). Sections (4 μm) were cut and stained for haematoxylin and eosin. Images were captured using the AE2000 binocular microscope (Motic) at × 20 magnification with Leica Application Suite 3.6 software. The number of invading cell cohorts was counted using the ImageJ (NIH, 1.47v). In brief, bright-field haematoxylin and eosin images were changed to 8 bit type, and then converted into binary. The holes were filled and the non-invading layer was removed. The invading cohorts were counted and the total area was calculated by restricting the size analysis to the size interval of cohorts and circularity to 0–1. Total number of invading particles per field was presented as one data point.

### MLC-2 rescue and functional assays

To re-introduce MLC-2 expression in ATRA-treated PCSs; PSCs were treated with 1 μM ATRA for 10 days, and then transfected with 2 μg MLC-2 plasmid, (pEGFP-MRLC1, a gift from Tom Egelhoff, Addgene plasmid #35680) for 4 h using JetPRIME reagent (1:3 DNA:jetPRIME ratio (w/v)) and JetPRIME buffer (Polyplus, 114-15). During transfection, cells were cultured in 2 ml media without ATRA to exclude the possibility of it affecting the transfection efficiency. After transfection, culture media was changed with 2 ml media containing ATRA. Mock transfection was done using JetPRIME reagent and buffer only (that is, without DNA) and the cells were otherwise treated the same way as the transfection group. Functional assays were done 48 h after transfection. To study the effect of MLC-2 overexpression on ECM remodelling, MLC-2 overexpressing ATRA-treated PSCs and mock transfection group were trypsinised and 500,000 cells were embedded in 80 μl Collagen-I Matrigel mixture gels (4.5 and 2 mg ml^−1^ final concentration, respectively). After 1 h incubation at 37 °C on 2% BSA-treated wells of 96-well plate, gels were covered with ATRA-containing media and left to be remodelled 3 days at 37 °C. Gel contraction was calculated as % reduction in the gel surface area.

### G-LISA assay for Rac1 and RhoA

The intracellular amounts of Rac1-GTP and RhoA-GTP were determined using the G proteins-linked assay (G-LISA) (Cytoskeleton, Inc., Denver, CO, USA) according to the manufacturer's instructions. In brief, cells were washed with cold PBS and homogenized gently in ice-cold lysis buffer. A measure of 20 μl was removed for protein quantification to adjust sample concentration to 0.5 mg ml^−1^. After adding an equal volume of binding buffer, triplicate assays were performed using 1.5 μg protein per well. Samples were incubated for 30 min and then washed three times with washing buffer. Antigen-presenting buffer was added for 2 min before removal; samples were then incubated with 1:250 dilution of anti-Rac1 and anti-RhoA antibodies, respectively, at room temperature for 45 min, washed three times and incubated with secondary antibodies for another 45 min. HRP detection reagent was added and signal was read by measuring absorbance at 490 nm using a microplate spectrometer.

### Migration assay

Cells were cultured on 35 mm glass-bottomed dishes pre-coated with 10 μg ml^−1^ FN and grown to a confluence of 95–100% in culture media DMEM with 2% FBS containing DMEM. On reaching confluence, ATRA treatment was applied to the treated population for 10 days before scratch assay measurements. A linear scratch was applied to the cell monolayer with a sterile 100 μl plastic pipette tip. Cellular debris was removed from the dish through a wash with DMEM before measurement. Scratch assays were kept at 37 °C and images taken along the length of the scratch were obtained with phase contrast microscopy with a 10 × objective. Images were taken at time intervals of 0, 24 and 48 h. Images were analysed in a custom program (Matlab) to detect the cell-free area in the scratch and the percentage change was calculated to quantify the wound closure.

### Statistical analysis

All statistical analyses were conducted with the Prism graphical software (GraphPad, Software). Data were generated from multiple repeats of different biological experiments to obtain the mean values and s.e.m displayed throughout. *P* values have been obtained through *t*-tests on paired or unpaired samples with parametric tests used for data with a normal distribution and non-parametric tests conducted via the Mann–Whitney test where data had a skewed distribution. Significance for the *t*-tests was set at *P*<0.05 where graphs show significance through symbols (*P<0.05; **P<0.01; ***P<0.001).

### Data availability

The authors declare that data supporting the findings of this study are available within the article and its Supplementary Information files.

## Additional information

**How to cite this article:** Chronopoulos, A. *et al.* ATRA mechanically reprograms pancreatic stellate cells to suppress matrix remodelling and inhibit cancer cell invasion. *Nat. Commun.* 7:12630 doi: 10.1038/ncomms12630 (2016).

## Supplementary Material

Supplementary Information Supplementary Figures 1-14 and Supplementary Methods

Supplementary Movie 1 Heat map videos of activated control (left) and ATRA treated (right) PSCs exerting forces on the elastic micropillars. Colour bar represents the forces in pN. This 10 second video represents 10 minutes of real time. The original video was recorded at 0.5fps. To reduce video size every 5th frame was used and the final video recorded at 6fps. The video is therefore 60 times the speed of real time.
