# Supplementary material for: ATRA mechanically reprograms pancreatic stellate cells to suppress matrix remodelling and inhibit cancer cell invasion
Source: Nat Commun. 2016 Sep 7;7:12630. doi: 10.1038/ncomms12630 (PMC5023948; doi:10.1038/ncomms12630)
Supplement: Supplementary Information — Supplementary Figures 1-14 and Supplementary Methods [file ncomms12630-s1.pdf]

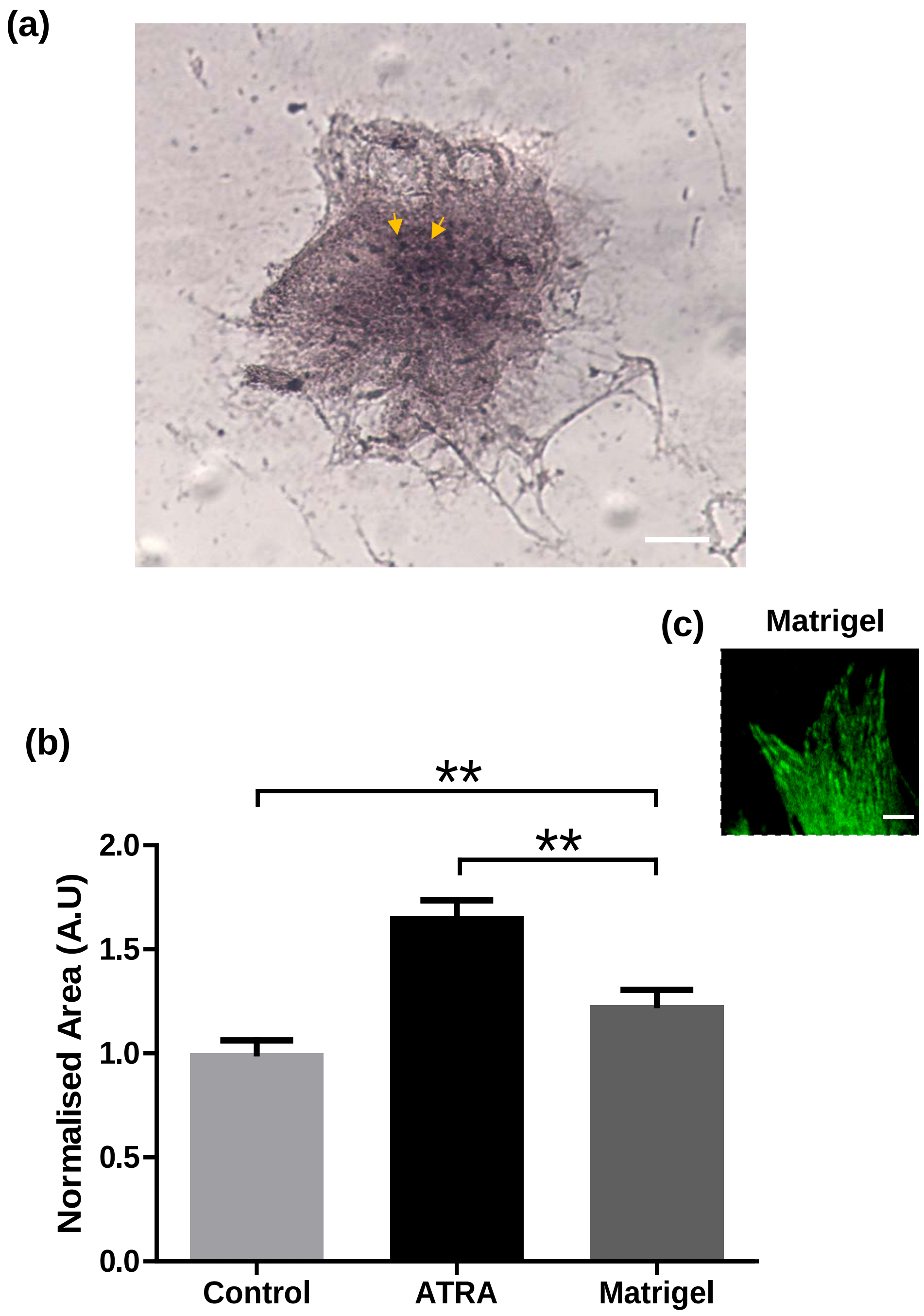

Supplementary Figure 1: Focal adhesion sizes in PSCs in the quiescent state induced by PSCs cultured on matrigel. (a) Oil red staining of PSCs grown for ten days on matrigel showing lipid containing vesicles indicative of quiescent phenotype, yellow arrowheads. **(b)** Quantification of focal adhesion sizes in PSCs control (activated by cultured on glass), ATRA treated PSCs cultured on glass, and quiescent PSCs cultured on matrigel. Histogram bars show mean ± SEM, n > 10, **p < 0.01 (one –way Anova with Tukey's post-hoc test). **(c)** Immunofluorescent images of paxillin containing focal adhesions in PSCs grown on matrigel. Scale bars in **(a)** and **(c)** are 25μm and 5μm respectively.

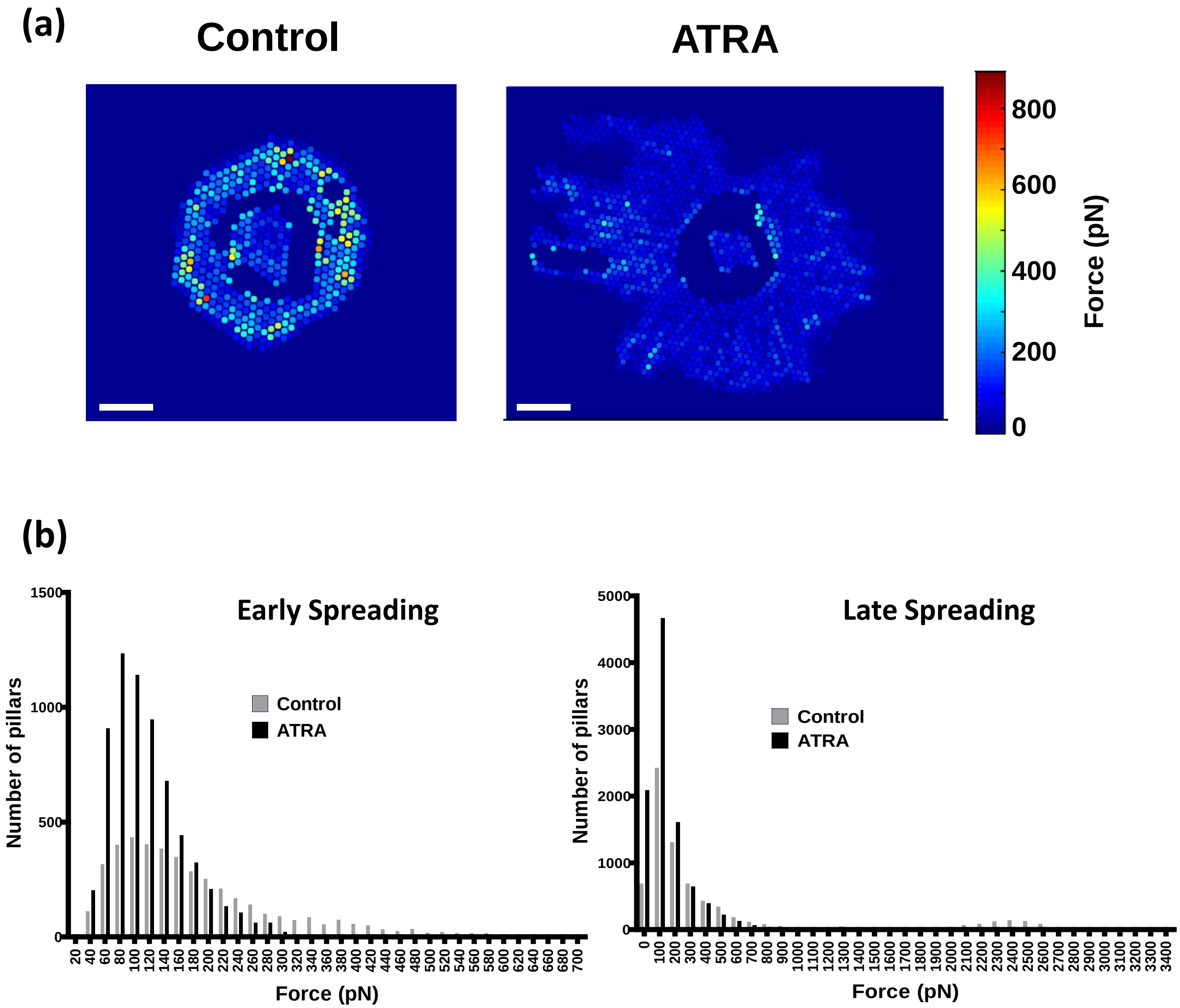

Supplementary Figure 2: Heat maps for the PSCs on top of the pillars. (a) Forces were calculated from the maximum pillar displacement through a custom-built tracking algorithm. Maximal forces for control PSCs were observed in regions located at the cell periphery, where the magnitude of the force in these regions saw a four-fold increase closer to the centre of the cell. ATRA treated PSCs displayed lower overall forces across the whole spreading area. Numbers in the scale represent force values in picoNewton. Scale bar 10 μm. **(b)** Frequency distribution for forces applied on each pillar for early and late spreading.

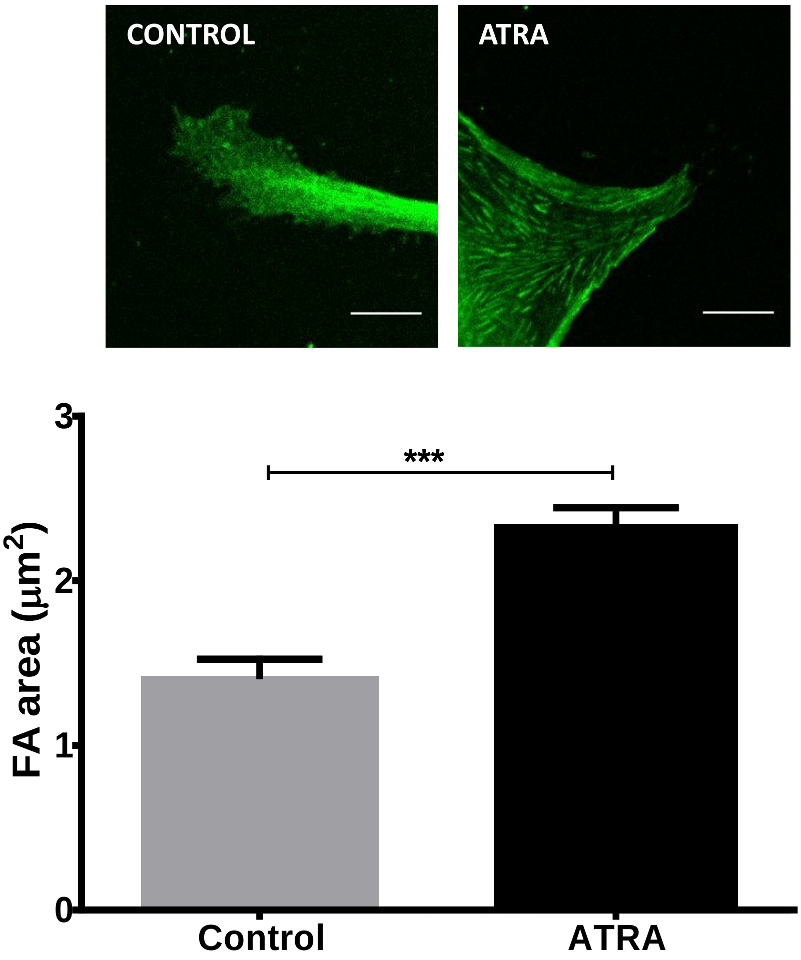

Supplementary Figure 3: : Quantification of FA areas in PSCs seeded on 12 kPa acrylamide gels. ATRA treated PSCs displayed significantly larger FAs (mean ± SEM 2.3212 ± 0.1180, n=76) than Control PSCs. (Mean ± SEM 1.423 ± 0.1007, n=52) $p < 0.0001$ (t-test). Images show paxillin staining. Scale bar 10 μm

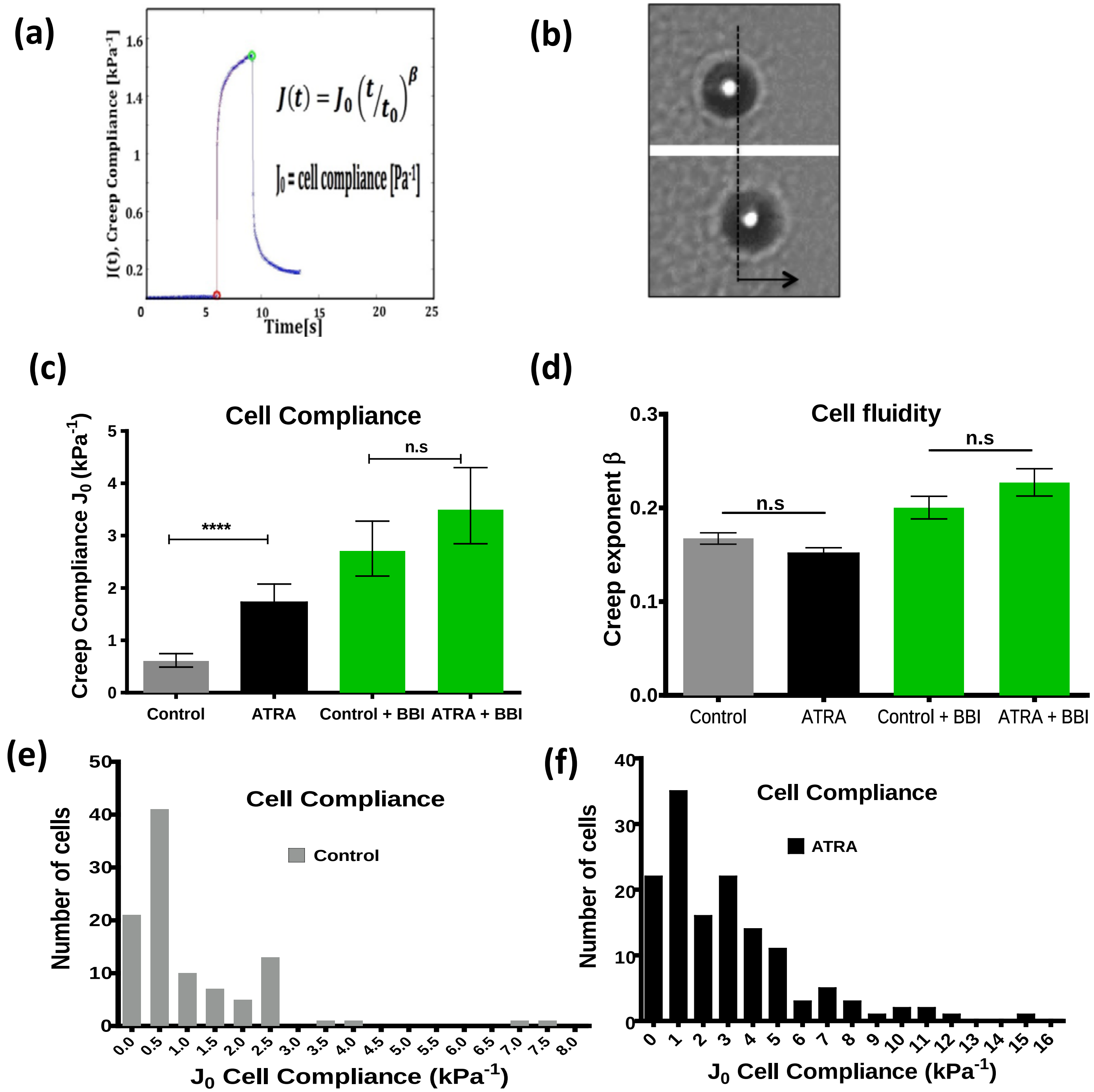

Supplementary Figure 4: Characterization of cell mechanics in PSCs using microrheology. (a) Representative trace used for the quantification of PSCs cell compliance (inverse of stiffness). **(b)** Images of the bead displacement before (top) and after (bottom) the force application. To examine the cytoskeletal stiffness of pancreatic stellate cells, we recorded their viscoelastic creep behaviour which refers to the amount of cell deformation under constant load. Using magnetic tweezers we applied constant mechanical tension (3 nN for 3 sec) onto FN-coated magnetic beads attached to the cytoskeleton of single cells via integrin-containing focal adhesions, and tracked the bead displacement under 20 frames per second. **(c)** Quantification of PSCs compliance. ATRA induced a significant softening of the cells evident by a ~2.8 fold increase in the cell compliance compared to PSC control (1.7 kPa^{-1} for PSC ATRA vs. 0.6 kPa^{-1} for PSC Control). No significant differences observed when BBI was used to block actomyosin cell contractility (3.4 kPa^{-1} for PSC ATRA vs. 2.7 kPa^{-1} for PSC Control). **(d)** Quantification of PSCs fluidity, characterized by the dimensionless value β, representing values from 0 to 1, pure elastic $\beta=0$ and pure viscous $\beta=1$. No differences were observed between Control and ATRA PSCs. PSCs displayed values close to pure elastic. **** $p < 0.0001$. Mann-Whitney test used in panels c and d. **(e, f)** Histograms with frequency distribution for Control and ATRA PSCs compliance. In all cases, more than 100 cells were analysed in 5 experimental replicates. Bars and error bars represent geometric mean and 95% confidence intervals respectively.

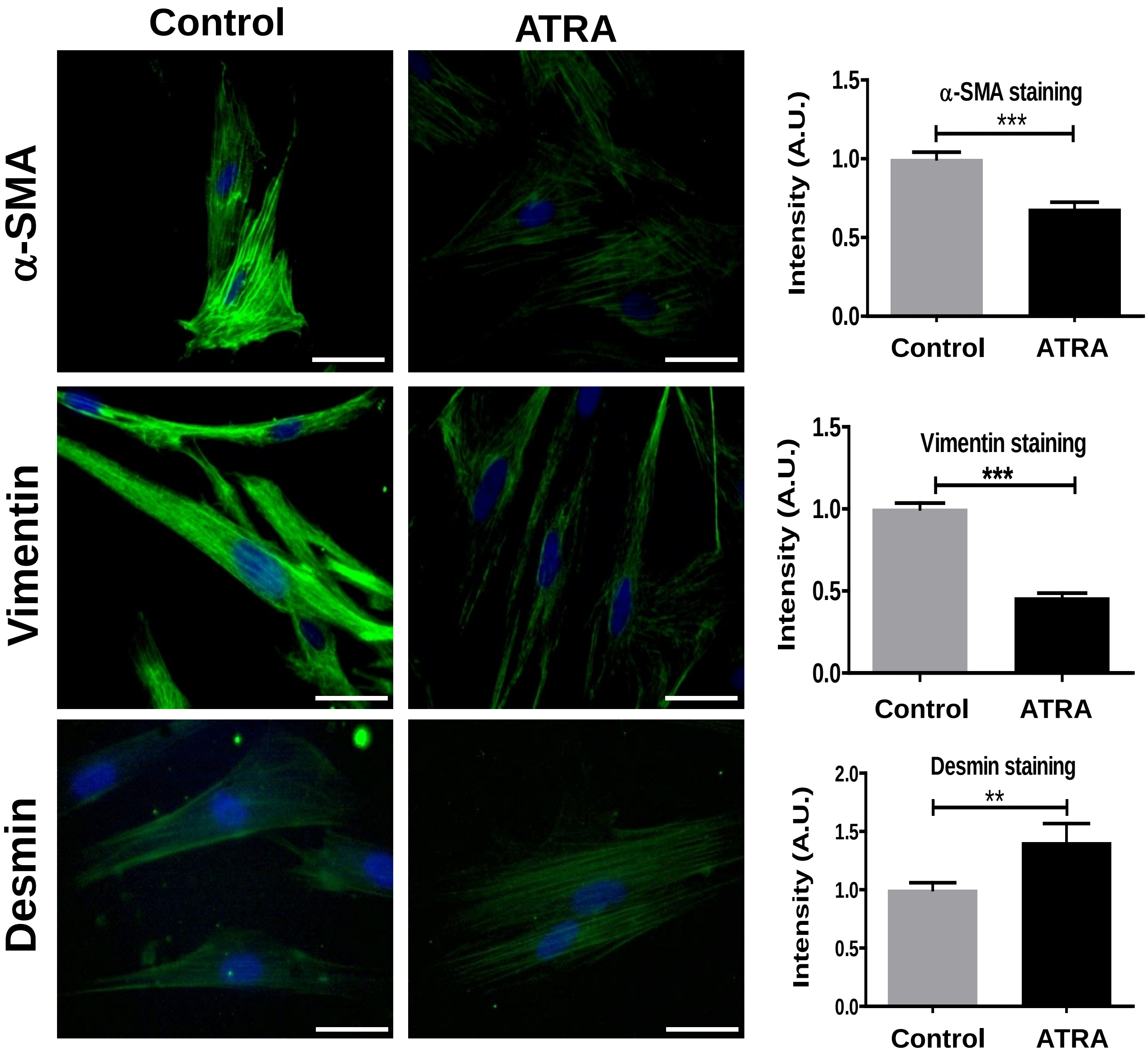

Supplementary Figure 5: ATRA induces a quiescent-like phenotype in PSCs. Immunofluoresce images for the alpha smooth muscle actin, vimentin (markers for myofibroblast phenotype) and desmin (marker for PSCs). The intensity of the staining was calculated by measuring Mean Fluorescent Intensity (MFI) of cell area by ImageJ. Scale bars 50μm. Histograms represent mean mean ± SEM. **, $p<0.01$; ***, $p<0.001$ (t-test). n>30 cells per condition collected in 4 different experiments.

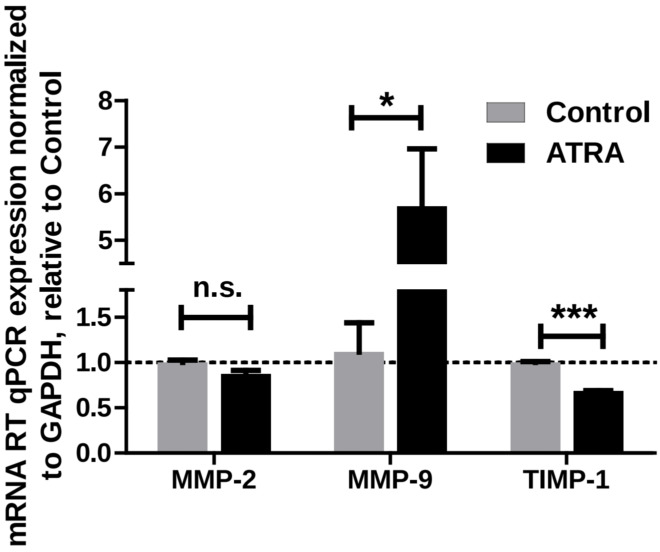

Supplementary figure 6: ATRA treatment up regulates MMP-9 and down regulates TIMP-1 expression in PSCs, but has no effect on MMP-2 levels. mRNA RT qPCR levels of MMP-2, MMP-9, and TIMP-1 in PSCs expressed as mean ± SEM; values are normalised to GAPDH and presented relative to control, n=3 experimental replicates, * $p < 0.05$ and ***$p < 0.001$ (t-test)

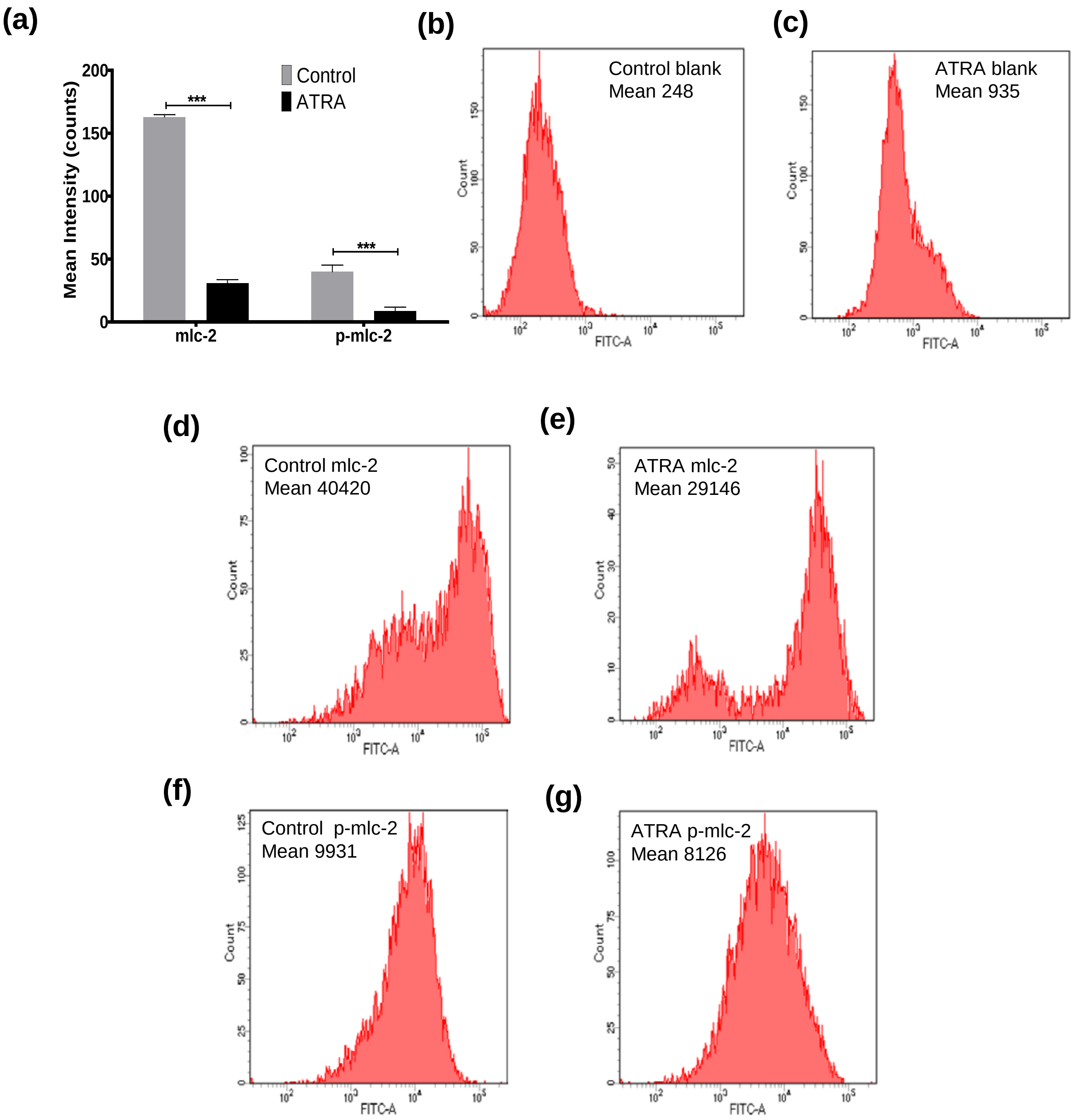

Supplementary Fig. 7: Flow cytometry analysis of mlc-2 and p-mlc-2 expression levels in control and ATRA treated PSCs. (a) ATRA treated PSCs show a five-fold decrease in both MLC-2 and p-MLC-2 levels with regard to control PSCs. Each value has been normalised against the respective blank (Control MLC-2 mean ± SEM 163.50 ± 1.79, ATRA MLC-2 mean ± SEM 31.55 ± 2.63, Control p-MLC-2 mean ± SEM 40.25 ± 5.16, ATRA p-MLC-2 mean ± SEM 8.74 ± 3.11) *** $p < 0.0001$ (t-test). **(b, c, d, e, f, g)** Representative histograms for control blank, ATRA blank, control MLC-2, ATRA MLC-2, control p-MLC-2, ATRA p-MLC-2, respectively. Data collected in 3 different experiments

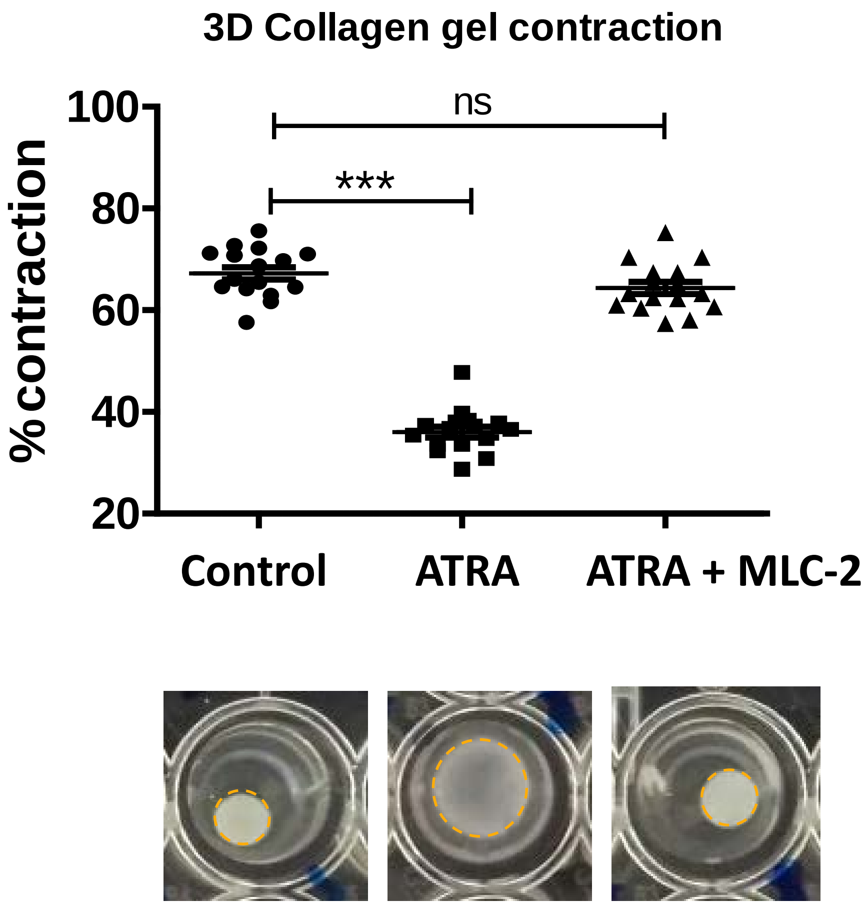

Supplementary Figure 8: Quantification of 3D collagen gel contraction expressed as a % change of gel area. ATRA treated PSCs contracted the gel at a significantly smaller extent (mean ± SEM 36.01 ± 1.07) than Control PSCs. (Mean ± SEM 67.27 ± 1.20) $p < 0.0001$ (Anova with Tukey's post-hoc). ATRA treated PSCs rescued with MLC-2 display no significant differences in their ability to contract the gel with regards to control PSCs (Mean ± SEM 64.37 ± 1.21). Each data point represents a gel, 4 experimental replicates. Dotted yellow lines represent the gels contours.

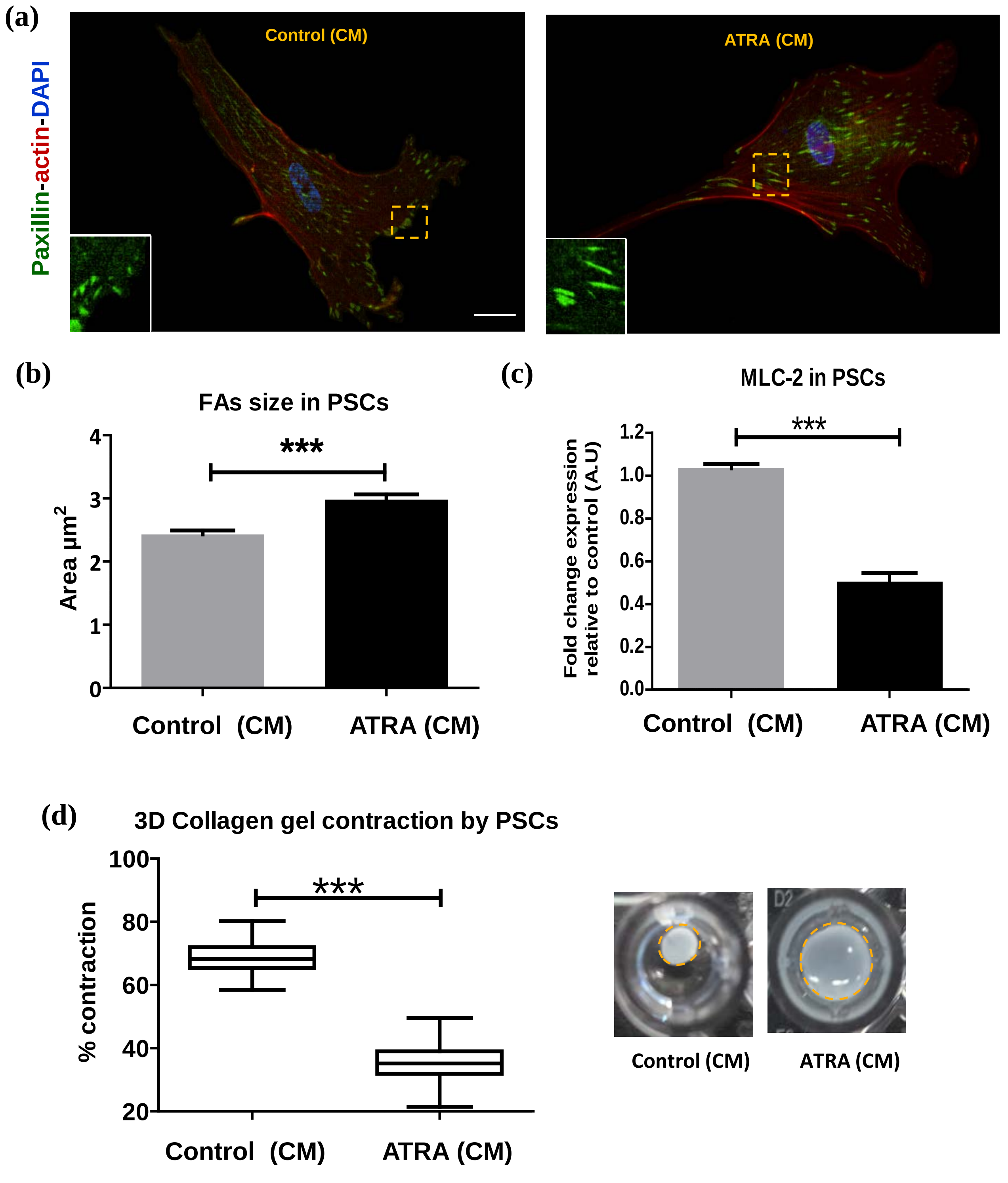

Supplementary figure 9: ATRA treatment increases focal adhesion sizes and decreases MLC-2 levels and force induced gel contraction in PSCs previously exposed to AsPC1 cancer cells conditioning media (CM). (a) Immunofluorescent images of PSCs showing paxillin containing focal adhesions (green), actin (red), and nucleus (blue). Scale bar represents 20 µm. **(b)** Histogram shows quantification of focal adhesion sizes . Values are expressed as mean ± SEM. **(c)** mRNA levels for MLC-2 in PSCs previously exposed to conditioning media (CM) before vehicle or ATRA treatment. Values are expressed as mean ± SEM. n > 10 cells, 3 different experiments **(d)** Images show 3D gel contraction by PSCs. In the box-and-whisker plot, the central box represents values from the lower to the upper quartile. The middle line represents the mean. The vertical line extends from the minimum to the maximum value. Control and ATRA 11 and 12 gels, respectively assessed over multiple experiments. Dotted yellow lines represent the gels contours. In all panels, ***$p < 0.001$ (t-test).

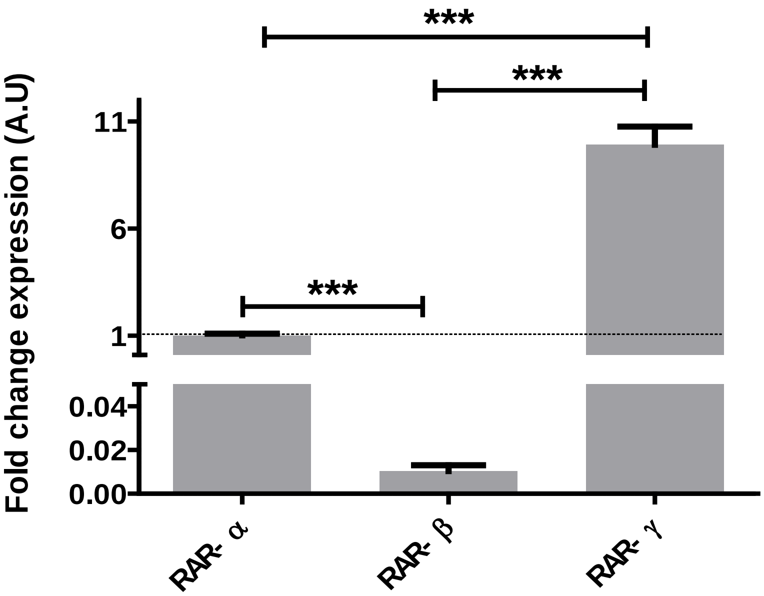

Supplementary Figure 10: Retinoic Acid Receptors (RAR) are expressed in PSCs. mRNA levels of retinoic acid receptors alpha, beta, and gamma (RAR-α, RAR-β, and RAR-γ, respectively) in PSCs. Fold changes in expression calculated and normalised to expression of RAR-α. Histogram bars represent mean ± SEM of three independent experiments. *** $p < 0.001$ (Anova and Tukey's post-hoc test).

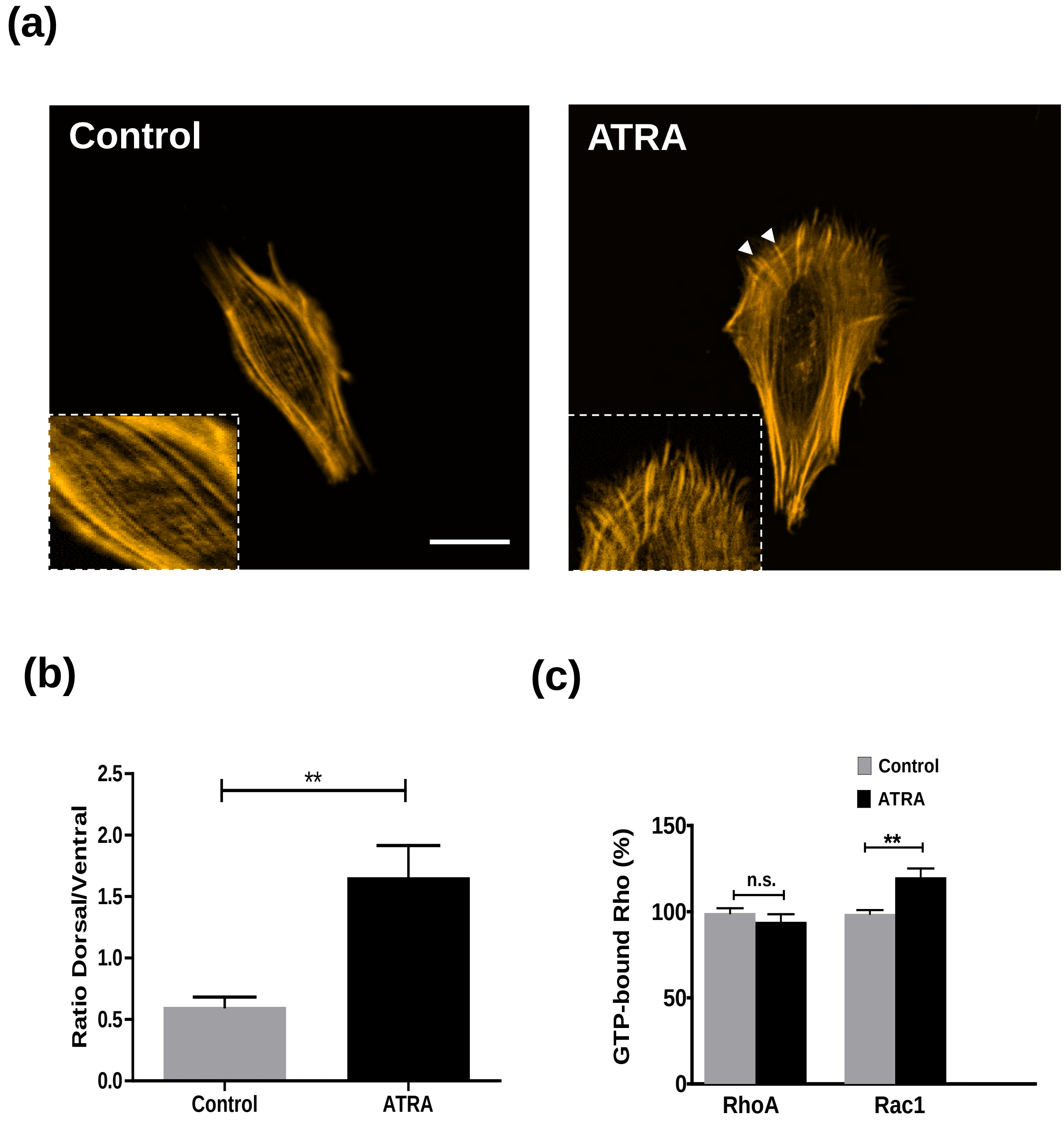

Supplementary Figure 11: Dorsal stress fibers formation and RhoA & Rac1 activation levels in control and ATRA treated PSCs. (a) Images of F-actin stress fibers on PSCs. Scale bar 25 mm. Dorsal or radial stress fibers (filled arrowhead) form in the lamella and elongate perpendicular from the leading edge towards the interior of the cell in ATRA treated PSCs, n > 15 cells per condition **(b)** Quantification of the dorsal/ventral stress fibres ratio in PSCs. **(c)** Quantification of the levels of activation of RhoA and Rac1. Percentages are relative to control values. In all cases histogram bars represent mean ± SEM of three independent experiments. ** $p < 0.01$ and n.s. no significant differences (t-test).

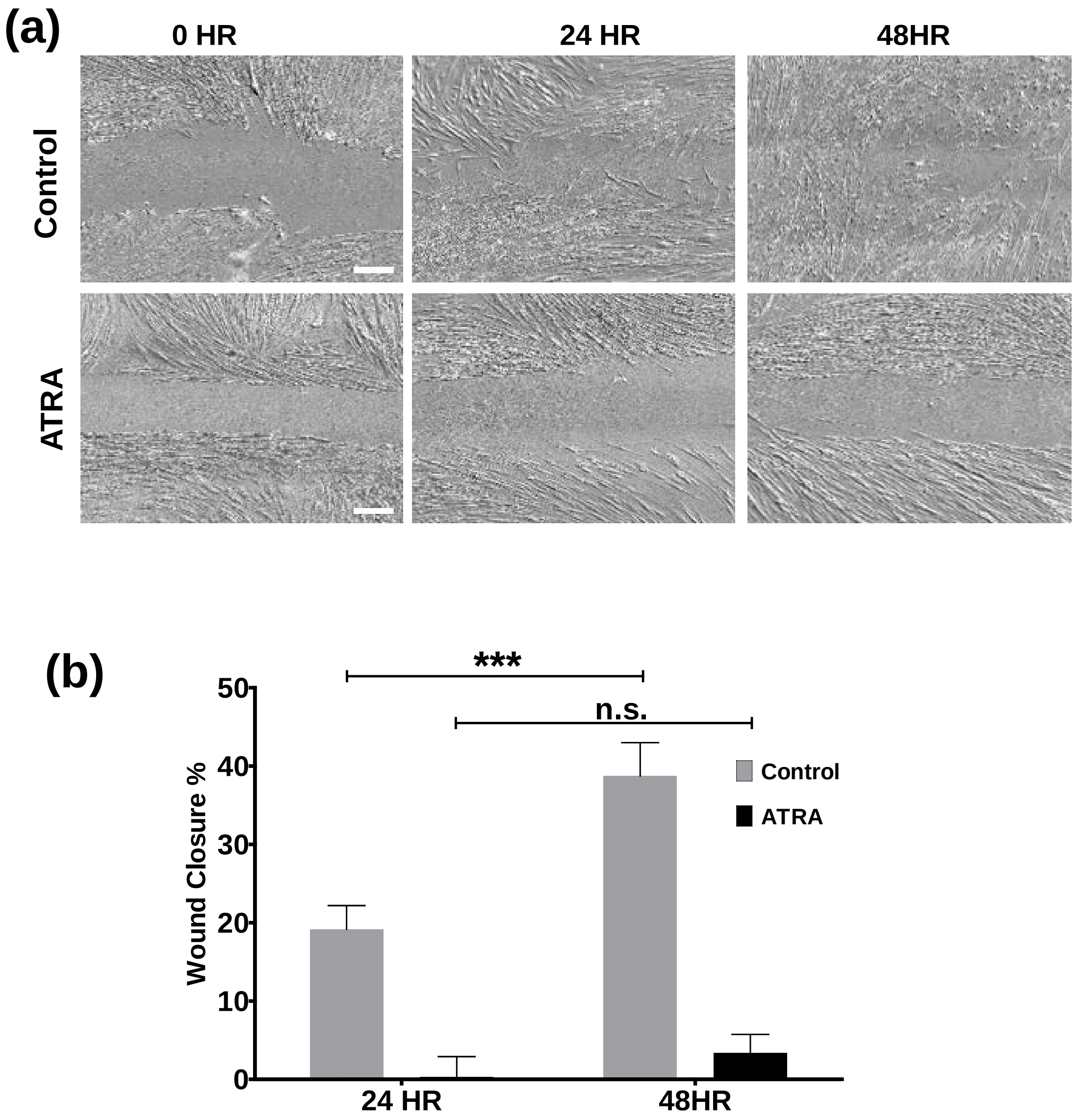

Supplementary figure 12: ATRA treatment impedes PSCs migration. (a) Bright field images of PSCs migration. Scale bar 200 mm. **(b)** quantification of % wound closure given by the % change in the cell free area against 0 h, 3 experimental replicates. All results are expressed as mean ± SEM. *** $p < 0.001$ and n.s. no significant differences (t-test) .

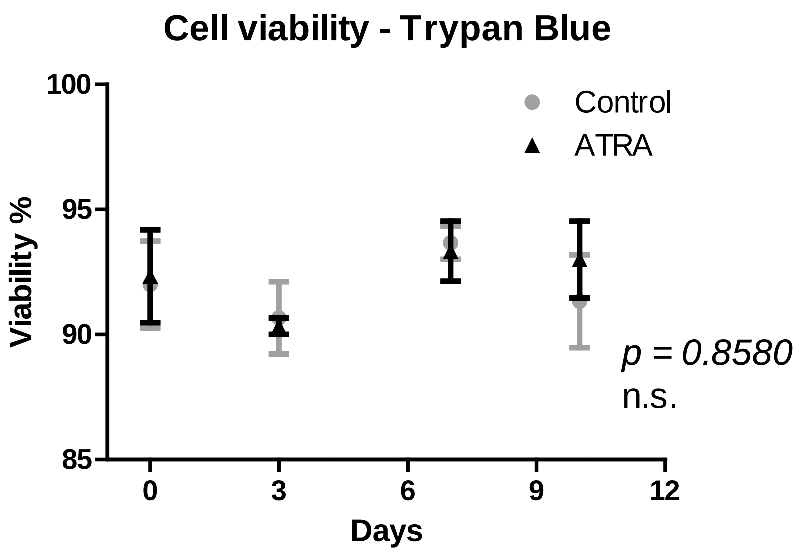

Supplementary Figure 13: ATRA treated does not change cell viability significantly. Trypan Blue was used to quantify number of dead cells from days 0 to 12 since the beginning of the ATRA treatment. No significant differences were observed between the untreated control and ATRA treated PSCs, and both groups showed a viability around 90%. p = 0.8580 (t-test). Three independent experiments

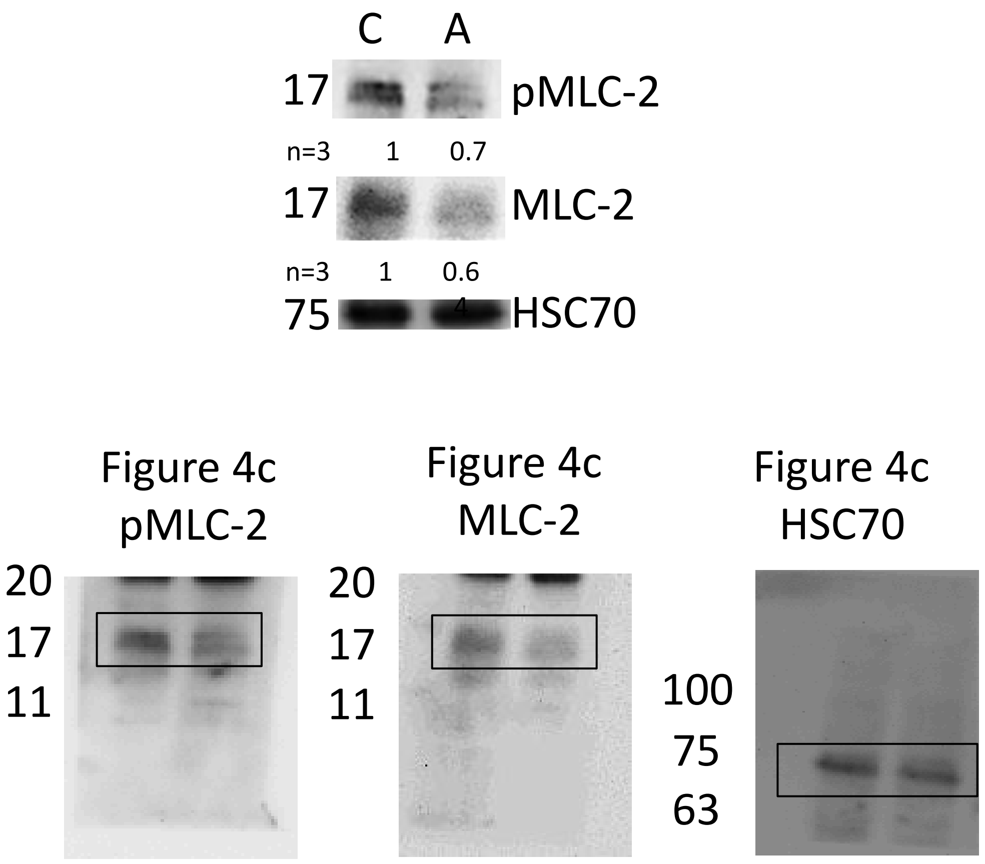

Supplementary Figure 14: Uncropped images of the Western Blot membranes corresponding to experiments presented in figure 4c.

Supplementary methods

Micropillar fabrication: High-resolution photolithography was used to create molds containing arrays of holes on a silicon substrate. In the fabrication of the arrays, the photoresist was patterned using a 5x reduction autostepper and the desired hole depths for the pattern were achieved through a C_4F_8/SF_4-based deep reactive ion etch. After removal of the photoresist the silicon molds were cleaned with piranha solution as well as a 1min O_2 plasma clean followed by silanisation with (tridecafluoro-1,1,2,2,-tetrahydrooctyl)-1-trichlorosilane overnight under vacuum. Polydimethylsiloxane (PDMS) was mixed with its curing agent (Sylgard 184; Dow Corning) at 10:1, poured over the silicon molds and subsequently placed under vacuum for 30mins, to remove air and ensure PDMS penetrated the full depth of the holes. The PDMS was cured at 70°C for 12h to achieve a Young's modulus of 2.0 ± 0.1MPa, and the PDMS was thus peeled off while immersed in ethanol.
The pillar substrates were comprised of a hexagonal array with centre-to-centre distances of twice the pillar diameter to achieve a constant area density. The pillar bending stiffness (k) was calculated through Euler-Bernoulli beam theory:

$$k = \frac{3}{64}\pi E \frac{D^4}{L^3}$$

where D, L and E are diameter, length and Young's modulus of the pillars, respectively. The pillar dimensions used for this study were 6µm for pillar length and 1µm for the pillar diameter leading to a pillar stiffness of 1.36 nN/µm.

Magnetic tweezers experiments: A magnetic tweezers device was used to apply tensional forces to fibronectin-coated beads bound to integrin receptors on the surface of cultured cells, and to measure stress-induced bead displacements. Tensional magnetic forces were induced by a high gradient magnetic field that was generated by an electromagnetic (solenoid-enclosed) microneedle with a pole tip (~15µm radius of curvature) that was microfabricated by mechanical milling and polishing with fine grit sandpaper (#4000 grade). The tip of the electromagnetic tweezers was positioned relative to a 4.5 µm diameter superparamagnetic bead (Dynabeads, Life Technologies) bound to cell surface integrins within the working distance of the 40x objective on an inverted microscope (Nikon Eclipse Ti-B) using an electronic micromanipulator (Eppendorf). Images of the bead position were recorded with an sCMOS camera at 20 frames/sec in brightfield mode. A computer control amplifier (TS200, Accel Instruments, CA) was used to provide electric current to the electromagnetic tweezers, and LabVIEW software was used to program the desired voltage waveform that was digitised and sent to the input terminals of the amplifier. A custom-built MATLAB algorithm was then used to analyse the image sequences and track bead displacement by following the intensity-weighted centroid of the bead across all captured frames. To calibrate the applied magnetic force, 4.5 µm beads were magnetically pulled through a silicone oil solution of known viscosity and their velocity was tracked across successive frames to obtain the force-distance relationship at various currents using the Stokes formula $F=6\pi\eta rv$, where η=viscosity of silicone oil, r = radius of bead, v = bead velocity.

Cellular microrheology: To characterize the mechanical properties of PSCs, we used magnetic tweezer microrheology to measure cell deformation in response to magnetically generated forces. Superparamagnetic 4.5 µm epoxylated beads (Dynabeads, Life Technologies) were coated with fibronectin (40 µg per 8 x 10^7 beads) and incubated with adherent cells for 30 minutes, prior to measurements, to allow integrin binding and provide a mechanical link between the bead and the cytoskeleton. The unbound beads were removed by

multiple washing with PBS. The experiments were performed at 37°C, 5% CO_2 and 95% humidity in DMEM containing 2% FBS in a microscope stage incubation chamber. A viscoelastic creep experiment was conducted by applying mechanical tension onto single beads bound on the apical surface of the cells with a constant pulling force (F_0 = 3nN) for 3 seconds generated by the magnetic tweezers. The viscoelastic creep response of the cells was recorded by tracking the resulting bead displacement in brightfield (40x objective at 20 frames per second) that is indicative of the local cell deformation. The viscoelastic creep response J(t) of cells during force application followed a power-law in time $J(t) = J_0(t/t_0)^{\beta}$ with the prefactor J_0 representing cell compliance (J_0 = inverse of cell stiffness in units of kPa^{-1}) and the dimensionless exponent β representing cell fluidity with values ranging between $0<\beta<1$ pure elastic ($\beta = 0$) or viscous behaviour ($\beta = 1$) and with the reference time t_0 was set to 1 sec. The creep compliance J(t) of the cell is essentially the ratio ($\gamma(t)/\sigma_0$) of the localized cellular strain $\gamma(t)$ induced by the applied stress from the magnetic tweezers σ_0, with $\gamma(t)$ taken as the radial bead displacement normalised over the bead radius $\gamma(t)=d(t)/r$ and the applied stress as $\sigma_0 = F_0/4\pi r^2$ taken as the applied force normalised over the bead cross-sectional area.
